# Estimate of the revenue and economic contribution of the professional pest management industry in Georgia, United States

**DOI:** 10.1093/jee/toae029

**Published:** 2024-02-25

**Authors:** Jacob L Winkles, Benjamin L Campbell, Brian T Forschler

**Affiliations:** Department of Agricultural and Applied Economics, University of Georgia, Athens, GA 30602, USA; Department of Agricultural and Applied Economics, University of Georgia, Athens, GA 30602, USA; Department of Entomology, University of Georgia, Athens, GA 30602, USA

**Keywords:** urban pest management, economic impact, North American Industry Classification System (NAICS)

## Abstract

The Professional Pest Management Industry (PPMI) dates back over a century in the United States. Stakeholder calls for economic studies of the PPMI include, in the 1980s, the National Research Council, although there has been little to no progress on that topic. US Census and Bureau of Labor Statistics data indicate that revenue and employment for the PPMI in Georgia increased 117% from 1997 to 2021. We determined the revenue, employment, and economic contributions for the PPMI in Georgia, United States, using 2 methodologies applied to IMPLAN: primary survey data in combination with an open records request and publicly available Federal Economic data. Estimates of average revenue for the Georgia PPMI in 2021 were $833–$988 million, using the survey/open records and publicly available data, respectively. We utilized an economic modeling program, IMPLAN, to estimate the economic contributions by the Georgia PPMI in 2021 to be between $1.7 and $2.0 billion, with 13,000–14,000 jobs for the 2 respective data sets. We describe the methods and provide tutorials for other states or national organizations to follow to generate justifiable, comparable economic information on the PPMI. In addition, we discuss the unique position of the PPMI as heavily regulated by State Departments of Agriculture to advocate for including the PPMI economic values when reporting agricultural economic contributions.

## Introduction

The desire to control pests is a deep-seated part of the human psyche (Robinson 1996). The centuries-long migration of human populations to urban centers undoubtedly created an economic incentive for services related to the management of insect and vertebrate pests in the built environment (Hartnack 1943, Snetsinger 1983, Robinson 1996). Today, the professional pest management industry (PPMI) is a diverse collection of economic entities, from single operators to multinational businesses, spanning urban centers worldwide (GPMC 2023). Demand for services from this industry is based upon many factors, including public health concerns, invasive species, nuisance, weather, real estate market, and food or retail industry growth, but is independent of other factors such as interest rates and, therefore, often referred to as recession-proof (Harbison 2021).

The growing number of companies and demand for PPMI services during the last century in the United States motivated the founding of regional and state-level associations that supported regulation of the nascent industry (Snetsinger 1983). Those relationships resulted in both Federal and State statutes, codes, and laws that oversee aspects of pesticide manufacture, products, and use, in addition to consumer protection (ASPCRO 2023, US EPA 2023a, 2023b). The National Association of Exterminators and Fumigators, founded in 1933 to be a voice for professionalism and promotion of industry-wide standards of care, has changed names twice to National Pest Control Association in 1937 and National Pest Management Association in 1999 and continues to be a positive voice regarding regulatory, government affairs, technical assistance, verifiable training, public health, and consumer protection (Heal 1954, PMP Staff 2013). The Georgia Pest Control Association (GPCA), founded in 1950, provided a voice at the regional level, which assisted in the passage of the Structural Pest Control Act of 1955, which included the formation of the Georgia Structural Pest Control Commission (SPCC) in 1956. The SPCC is an oversight group within the Georgia Department of Agriculture (GDA) that regulates the PPMI in Georgia through statutes governing consumer protection, insurance, and pesticide training/certification requirements that are applicable to all PPMI businesses that operate in Georgia. Those regulations are codified in the Rules of the SPCC according to the Official Code of Georgia Annotated §43–45, Structural Pest Control Act (Georgia General Assembly 2021, 2023). Despite the involvement of the aforementioned industry and regulatory groups, there is a dearth of published economic data on the PPMI industry.

The call to illuminate the economics of pest management in the built environment goes back at least 4 decades (NRC 1980). Economic estimates can be used to compare goods and services, influence business strategic planning, and/or impact policymaker decisions. Economic figures associated with the PPMI have been met with skepticism by PPMI stakeholders because of the wide variability in estimates that are oftentimes published without attribution to data sources (Su 2002, Rust and Su 2012, PCT Staff 2019, 2020, NPMA 2023, Rust et al. 2023). Disparities in PPMI economic estimates can be, in part, ascribed to confusion around the terminology—revenue, impact, and contributions—used when discussing dollar values attributed to this service industry. The proprietary nature of small businesses, their willingness to share financial data, and the classifications used in federal economic databases can also contribute to a wide range of estimates (OMB 2022). Economic analyses are most frequently published as institutional reports outside the peer-review process (Khachatryan et al. 2021, English and Popp 2023, CAED 2022, Von Nessen 2022). There are published market analyses that utilize survey data or federal, publicly available data, but those analyses and estimates are fee-based—often outside the budget of small business owners, academic, and regulatory programs—yet, more importantly, the source of the data is not transparent even with memberships (IBISWorld 2023, Rust et al. 2023, Statista 2023, TBRC 2023).

We used publicly available sources and survey data, separately and together, to examine the economic contribution of the PPMI to the state economy of Georgia. The motivation for this study included filling a research gap by providing, for the first time, a reproducible methodology for economic assessment of the PPMI in one state, Georgia, with applicability for future surveys at the State or National level. Our specific objectives were to: (i) Clearly define the terms revenue, contributions, and impact in regard to PPMI economic estimates. (ii) Provide a template for locating economic data from public record sources at the state and federal levels. (iii) Compare and contrast economic estimates based on 2 different data sets, publicly available and survey with open records request for the calendar year 2021.

We discuss the importance of the estimates obtained using different data sets while highlighting the need to use common, standardized definitions of economic effects. The quantifications detailed in this work are intended to serve as a resource for creating comparable baseline economic estimates across all PPMI state/national economies. Lastly, we argue because of the regulatory oversight and resulting relationships with State Departments of Agriculture, PPMI services should be included as an agricultural economic input to state economies to highlight an overlooked partner in entomology departments within land grant institutions.

## Materials and Methods

### Glossary of Definitions

#### Certified operator

An individual licensed for one or more GDA certification categories (Wood Destroying Organisms, Household Pest Control, Fumigation) to use pesticides or supervise PPMI services. All establishments must have at least one designated certified operator.

#### Direct effects

Data representing the inputs for generating gross revenue by an industry within a specified timeframe.

#### Economic contribution

An analysis of revenue (total output) generated by industry, including the sum of direct, indirect, and induced gross revenues within a defined timeframe for a specific region (city, county, state, or national).

#### Economic impact

An analysis of the change in revenue (direct output) of an industry (i.e., new business moving into the area, revenue-affecting policies, and natural disaster) within a defined timeframe for a specific region.

#### Establishment

All economic information collected for this assessment is based on data recorded by both State and Federal datasets at the level of an *establishment.* This is the baseline unit by which the GDA oversees the regulation of the PPMI industry. Establishment refers to an “office” or “branch office” in the PPMI vernacular. Establishments have been referred to as “companies” in GDA Structural Pest Control Division as well as PPMI association fact sheets and communication. It is not feasible to report PPMI economic data by company because a single company can operate out of multiple establishments as well as consist of several brands that include multiple establishments.

#### Indirect effects

Data representing money spent on the supply chain, vendors, and distributors by industry for a specified time frame.

#### Induced effects

Data representing the household spending by employees of the industry of interest within a specified timeframe.

#### Location quotient

The ratio of a specified industry’s employment to total employment in a specified region divided by the national ratio of the specified industry’s employment to total employment.

#### Market size

The total number of potential consumers of a service or product in a particular market segment and the revenue that this consumption can generate.

#### Output

Data generated representing gross revenue by industry for a specified time frame.

#### Registered employee

A licensed individual engaged in PPMI services that, in Georgia, are required, by regulation, to attain 70 h of experience prior to registration and 10 h of continuing education units to maintain registration.

#### Revenue

Income generated over a set period through the sale of goods or services without expenditure costs removed. Revenue and output are synonymous.

#### Value added

The money generated by labor income, other property income, and taxes on revenue for an industry within a specified timeframe.

### Data Collection

Two data sets were collected and analyzed to obtain estimates of economic contributions for the Georgia PPMI in the year 2021, the last year that Federal Economic data are available. The first data set encompassed gross revenue provided from anonymous in-person surveys and employment data obtained through an open records request within the GDA Structural Pest Division (SPD) (Georgia General Assembly 2016). The second data set was mined by accessing publicly available revenue data using the quinquennial Economic Census (EC) 1997–2017 from the United States Census Bureau and employment data obtained from Bureau of Labor Statistics’ (BLS) Quarterly Census of Employment and Wages (QCEW) (USCB 1997, 2017, BLS 2021).

### Economic Modeling

There are a number of economic models available for determining downstream activity. In this study, we employed the IMPLAN economic modeling program for the economic contribution analysis of the PPMI in Georgia using both datasets (IMPLAN 2021 Data). That modeling program relies on Social Accounting Matrices using an expanded input–output analysis to determine transactions between entities in a specified region, city, county, or state (Mainar-Causpé et. al 2018, Miller and Blair 2022). The IMPLAN software has an extraction-based economic contribution analysis function that uses embedded data from various industry sectors but places the PPMI in the industry group, Services to Buildings and Dwellings, where it represents approximately 15% of the data (Clouse 2023). Therefore, a 2-event approach was utilized where we set the Industry Impact Analysis event using total output (revenue) and total employment coupled with an Industry Contribution Event set at $1 to constrain feedback linkages within the model (Miller and Blair 2022).

### Data Set 1

In-person, anonymous surveys collected data for the 2021 calendar year from 2 groups: business owners and employees. The business owner survey asked for general company information, including number of employees, revenue, and expenses (Supplementary Material S1). The employee survey requested information on employee-related matters such as company size, compensation, and offered benefits (Supplementary Material S2).

The surveys were distributed at 2 State Association PPMI conferences: the Certified Pest Control Operators of Georgia (CPCO of GA) and GPCA. The first survey was distributed at CPCO of GA’s fall conference with 325 attendees on 25th and 26th October 2022, in Macon, Georgia, and the second at GPCA’s winter conference with 513 attendees on 10th–12th January 2022, in Athens, Georgia. The second survey asked respondents if they previously completed this survey, and respondents who answered yes were removed from the sample. Conference attendees were incentivized to return completed surveys with a chance to win 1 of 4 raffle prizes at the end of the conference. The survey response rate for CPCO of Georgia’s was 28.6% (30 owners and 63 employees), and GPCA’s conference 24.0% (42 owners and 81 employees) for a total of 72 owners and 141 employees.

We employed a production-based approach to determine total revenue and employee numbers for imputation into IMPLAN. Revenue values from each business owner survey were divided by the number of employees to obtain revenue per employee per establishment. The establishment values were then averaged to obtain the average state-wide revenue per employee for the entire sample. Georgia’s total servicing employees for 2021 were mined from licensure datasets obtained from GDA’s SPD, and employees were only counted if employed at a Georgia-based establishment. Total revenue for the PPMI in Georgia was calculated by multiplying the average state-wide revenue per employee and the total number of servicing employees in the state.

### Data Set 2

We used the historical annual percentage growth and future value approach to configure a forecast-based total revenue estimate for this data set. Data were obtained from EC surveys from 1997 and 2017; ECs categorize industries using the 6-digit North American Industry Classification System (NAICS) code with PPMI as code 561710. EC surveys were appropriate for this analysis because they collect data at the establishment—not company-based—level. The 1997 revenue value was inflated, prior to input into the model, to a baseline 2017 $USD with the Consumer Price Index (CPI) baseline equation; thus, a compound annual growth rate equation (CAGR) equation determined the real compound annual growth rate percentage, 3.94%, for that 20-year period. Determination of the 2021 revenue value utilized a future value equation with the present value equated to 2017 revenue. The future revenue value was inflated to 2021 $USD from the 2017 $USD baseline using the CPI inflation-adjusted equation to add the effects of inflation for point-in-time accuracy. The numbers for the 2021 employment values inputted into IMPLAN for the PPMI in Georgia came from the QCEW.

The pertinent equations for the estimation of revenue inputs based on the quinquennial-published EC data for intervening years are listed below:

**Consumer Price Index (CPI) Baseline Equation** (BLS 2023b)


$$\begin{array}{l} \begin{array}{lll} & \frac{CPI\;of\;baseline\;year}{CPI\;of\;year\;of\;interest}*Revenue\;estimate\;for\;year\;of\;interest\; & =Real\;revenue\;for\;year\;of\;interest\;in\;baseline\;year\; \end{array} \end{array}$$

**Compound Annual Growth Rate Equation** (Horngren et al. 2014)


$$\begin{array}{l} CAGR={\left(\frac{V_{Final}}{V_{Begin}}\right)}^{\frac{1}{t}}-1 \end{array}$$

where

*CAGR* = compound annual growth rate

*V*_*Final*_ = final value

*V*_*Begin*_ = beginning value

*t* = time in years

**Future Value Equation** (Horngren et al. 2014)


$$\begin{array}{l} Future\;value\;\left(FV\right)=Present\;value\;\left(PV\right)*{\left(1+r\right)}^{n} \end{array}$$

where

*FV* = future value (estimated value for year of interest)

*PV* = present value (value of last available year)

*r* = annual real growth percentage

*n* = number of years

**Consumer Price Index (CPI) Inflation-Adjusted Equation** (BLS 2023b)


$$\begin{array}{lll} & \frac{CPI\;of\;year\;of\;interest}{CPI\;of\;baseline\;year}*Revenue\;estimate\;in\;baseline\;year\;value\; & =Nominal\;revenue\;for\;year\;of\;interest \end{array}$$

## Results

### Data Set 1

Average revenue per employee was calculated from the survey data at $102,176 per servicing employee with a 90% CI [$93,089–$111,262]. The other estimated value imputed for 2021 included total employment 8,154 per GDA open records response. The model calculated the total revenue for the PPMI in Georgia for 2021 to be $833 million, with an industry-wide contribution to the State economy of $1.7 billion and 13,000 jobs (Table 1).

**Table 1.** Summary of IMPLAN output describing estimated annual employment, wages paid (labor income), revenue minus intermediate business expenses (value added), and total money (output) generated for the industry (direct effects), supply chain (indirect effects), and employee spending (induced effects), respectively, along with totals for the PPMI in Georgia for 2021 (IMPLAN® model 2021). Total output represents the economic contribution of the industry to the state economy, and direct effects output the total annual revenue generated by the survey and public records request data sets.

	Employment	Labor income	Value added	Output
Direct effects	8,154	$283,132,138	$373,388,724	$833,443,105
Indirect effects	2,596	$172,678,512	$260,778,896	$477,167,684
Induced effects	2,262	$126,142,099	$234,915,637	$398,985,858
Total	13,012	$581,952,749	$869,083,257	$1,709,596,647

### Data Set 2

The inputs for IMPLAN from the 2021 QCEW included total employment (8,489). Annual revenue of $988 million USD came from the EC along with the forecast based on published 1997 and 2017 EC figures using the forecasting method. The average annual revenue per employee was calculated at $116,482. The PPMI was estimated, from the aforementioned public databases, to have contributed $2 billion and 14,000 jobs to the Georgia State economy for 2021 (Table 2).

**Table 2.** Summary of IMPLAN output describing estimated annual employment, wages paid (labor income), revenue minus intermediate business expenses (value added), and total money (output) generated for the industry (direct effects), supply chain (indirect effects), and employee spending (induced effects), respectively, along with totals for the PPMI in Georgia for 2021 (IMPLAN® model 2021). Total output represents the economic contribution of the industry to the state economy, and direct effects output the total annual revenue generated by the economic census and quarterly census of employment and wages data sets.

	Employment	Labor income	Value added	Output
Direct effects	8,489	$294,764,376	$408,595,994	$988,816,638
Indirect effects	3,275	$217,782,162	$328,894,378	$601,803,944
Induced effects	2,544	$141,845,587	$264,159,544	$448,654,679
Total	14,308	$654,392,125	$1,001,649,917	$2,039,275,263

IMPLAN separated the economic contributions by direct, indirect, and induced effects. Direct effects represented the revenue and employment values imputed for the PPMI, while IMPLAN configured labor income and value-added economics for both indirect and induced effects. Indirect effects describe estimated economic contributions resulting from business-to-business purchases of inputs (i.e., purchasing tax filing services, pesticides, uniforms, and spray equipment). Induced effects capture the contributions from wages spent from both direct and indirect employment numbers on goods and services such as food, utilities, and housing.

A comparison of the estimates generated by the 2 models showed they were within 16% of the higher estimate (Tables 1 and 2). The near proximity of the estimates indicated a reasonable approximation for the range of annual revenue generated by the PPMI in Georgia. It can be expected that the survey data, because it involved fewer inputs and a smaller sample size, would slightly underestimate values compared to the public-available data set. We, however, believe the public data set also likely underestimated total revenue and employment values because EC data does not account for nonemployer revenue, which are typically self-employed sole proprietorships one-man operators. Nonemployer revenue accounts for approximately 3% of the total revenue for the PPMI in Georgia (USCB 2017a, 2017b).

**Fig. 1.**
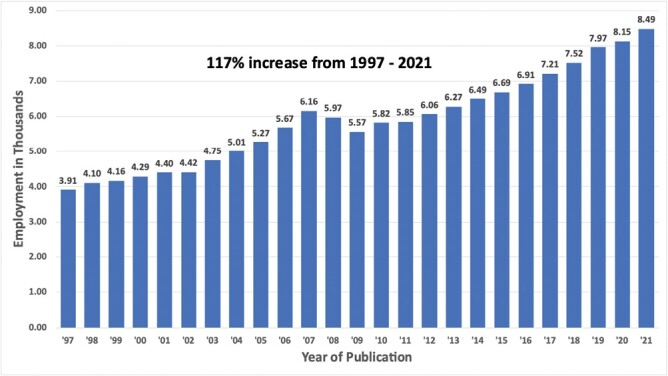
Graph, by year, of annual total employment numbers for the Professional Pest Management Industry in Georgia from 1997 to 2021 obtained from the quarterly census of employment and wages published by the Bureau of Labor Statistics (Supplementary Material S3) (BLS 2023c).

**Fig. 2.**
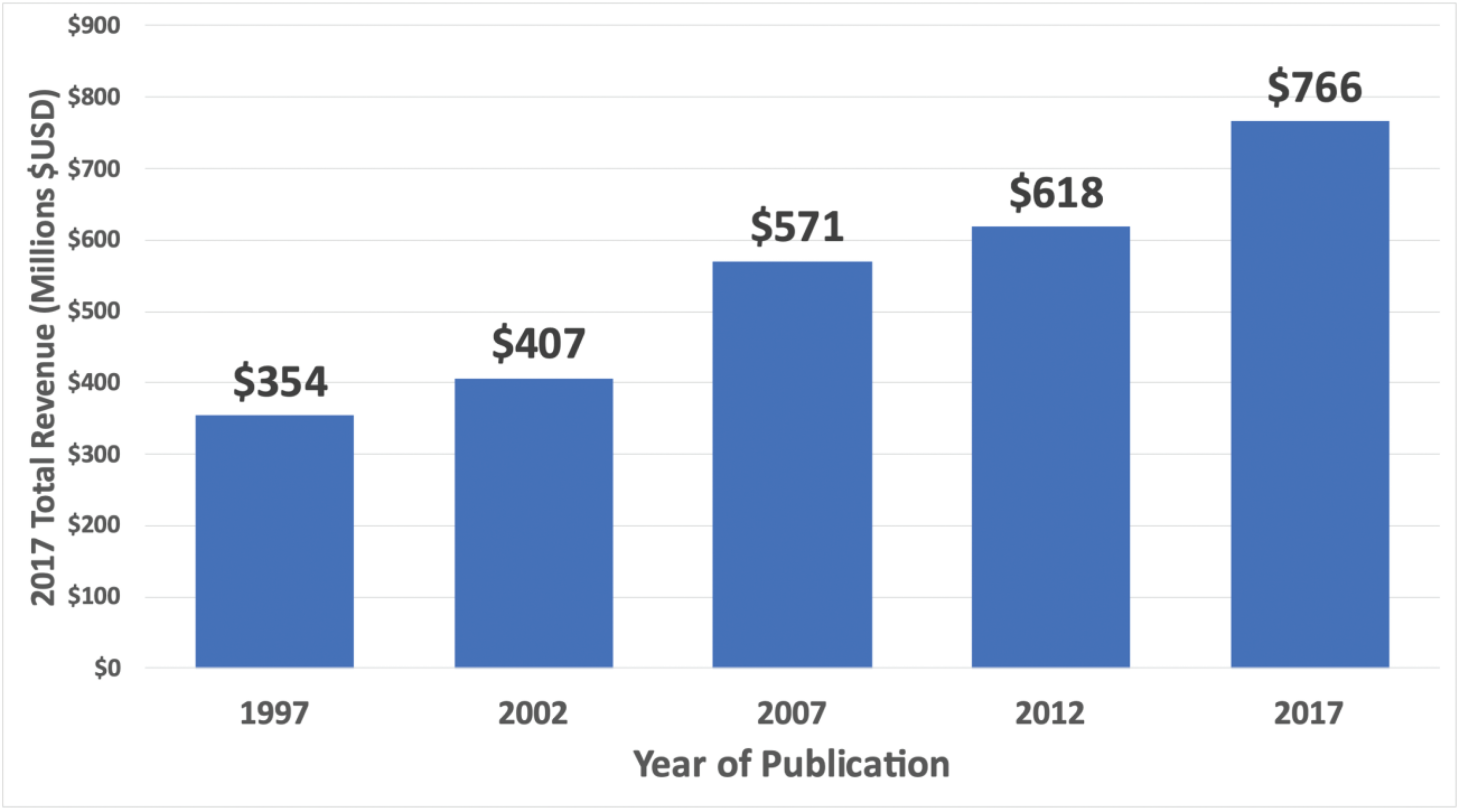
Graph of inflation-adjusted total revenue, by year of the census, from 1997 to 2017 for the Professional Pest Management Industry in Georgia from the United States Census Bureau’s quinquennial economic census (Supplementary Materials S4–S6) (USCB 1997, 2002, 2007, 2012, 2017a).

Historical data from the EC and QCEW provided valuable insight into the recent growth of the industry while validating our model estimates. The QCEW annual average employment for 1997 was 3,912; the total employment for PPMI in Georgia increased 117% from 1997 through 2021 (Fig. 1). Total revenue for the industry over a congruent 20-year period from 1997 to 2017 likewise increased 117% ($353,935,090–$766,294,000) after removing the effects of inflation (Fig. 2).

## Discussion

Economic analyses of pest management in agriculture and the built environment (PPMI) are important but often misinterpreted by economists and entomologists. Analyses of economic contributions are primarily conducted on resource-based as opposed to service-based industries (Jensen et al. 2020, Botta et al. 2021, English and Popp 2021). Service-based industries are equally, if not more important than resource-based ones, largely because regional economic contributions due to labor income are spent within a region. The US Department of Labor Statistics shows the service industry employs approximately 80% of all workers in the United States (BLS 2023a). Estimating the economic contributions from an intangible service is rather more difficult than tangible goods associated with manufacturing or agricultural commodities (Bryson and Daniels 2007). The need to estimate service sector economic contributions is highlighted by the 106% increase in the global service sector’s inflation-adjusted value added to global gross domestic product (GDP) from 1997 to 2021 ($26.67–$56.02 trillion), with the United States accounting for approximately 30% of that total value added while the United States agricultural sector is 5% of the global agricultural GDP (World Bank 2021a,b,c).

The PPMI industry in Georgia also experienced substantial growth from 1997 to 2021. Total employment and inflation-adjusted total revenue increased no less than twofold through that timespan. This substantial growth and concentration support that PPMI-provided services are a notable contribution to the Georgia state economy. The factors influencing that growth are beyond the scope of this study but are, in part, illuminated by the annual average employment location quotient (AAELQ). The AAELQ is a measure of the concentration of employment, and that metric for Georgia in 2021 was 1.98, meaning that PPMI employment is approximately twice as concentrated than the national proportion of PPMI employees, placing Georgia 3rd on that list (BLS 2021).

Georgia PPMI revenue estimations have been published 3 times in the past 21 years. Two of those estimates, in 2012 and 2020, published as informational leaflets, provided the same figure, $321 million (GPCA 2013, GPCA 2020, Supplementary Material S7). That information provided no methodology for the configured values, which when compared to QCEW data, underestimated revenues, opposed historical trends, and indicated that the PPMI experienced negative growth when accounting for inflation. In contrast, the published EC revenue showed the Georgia PPMI generated $579 and $908 million in revenue, respectively, for the 2012 and 2020 time frames while accounting for inflation (USCB 2012). The other previously published revenue estimate, $390 million in 2002, did provide a detailed methodology that relied on the estimated cost of services, and the estimated number of applications performed was an overestimate when compared to the 2002 EC revenue value of $298 million (USCB 2002, GPCA 2003, Supplementary Material S7). There has been one other publicly available economic contribution report on the PPMI in the Southeastern United States, but it lacked methodology surrounding the imputed revenue value for IMPLAN (Khachatryan et al. 2021). That report likely underestimated the imputed revenue value as the 2017 EC published revenue was $244 million greater (USCB 2017a, Khachatryan et al. 2021). The lower value imputed by Khachatryan et al. (2021) would indicate negative growth of −15%, −20% when accounting for inflation, while the national industry never indicated a decline in revenue from 1999 to 2021 (USCB 2017a, 2021). Describing a clear methodology and providing definitions for terms used in published economic estimates is vital for transparency, ease of replication, establishing a baseline to track future growth, and for other regions to follow suit and inevitable comparisons between published values.

Economic contribution and impact are not synonyms. Yet those terms are regularly juxtaposed in reporting economic data (Tootelian 2020, Von Nessen 2022). Watson et al. (2007) defines economic contribution, in the traditional economist lexicon, as the economic activity of an existing industry within a regional economy and economic impact as the measure of economic change resulting from an industry activity moving into or out of a region. The misappropriation of these terms stems, in part, from creating a marketable, appealing report title for stakeholders, policymakers, and industry controllers (Watson et al. 2007). The appropriate use of the 2 terms is vital to maintain transparency and accuracy when comparing economic activity within an industry or between industries.

One of the confounding influences that arise from inconsistent definitions for comparing economic estimates could be remedied with widespread access to public records. The Georgia PPMI data in EC and NAICS datasets are predicated on the GDA business license records which are based on registration overseen by the SPCC (Georgia General Assembly 2023). It is important to recognize that the NAICS rely on the same base unit as reported by the GDA, which is often confused in economic publications or reports by conflating the company with the establishment (branch offices). The hierarchal nature of the NAICS poses an issue regarding the ability of interested parties to locate PPMI data within a convoluted standardized economic statistic database. The hierarchical categorizations begin with sectors, the broadest classification in this system, which contain the following sequence: subsectors, then industry groups, then individual industries (Supplementary Material S8). Professional pest management offerings are placed within the Administrative and Support and Waste Management and Remediation Services (56) sector, more specifically, in the Administrative and Support Services subsector (561). Furthermore, Services to Building and Dwellings (5617) within subsector 561 contain 5 separate industry codes, which include professional pest management offerings (561710). This industry code, 561710, aggregates the following service offerings: bird proofing services, exterminating services, fumigating services (except crop fumigating), mosquito eradication services, pest inspection services, pest control services (except agricultural and forestry), and termite control services (OMB 2022).

An additional obstacle to estimating PPMI economic contributions includes a lack of useful historical data prior to 1997. The NAICS code replaced the Standard Industrial Classification code system, which aggregated the PPMI and the “disinfecting and deodorizing service industry” (USCB 2000). The NAICS, in 1997, placed the PPMI into a separate category called “janitorial service.” Therefore, PPMI economic data obtained from NAICS prior to 1997 do not accurately represent the PPMI due to statistical aggregation.

One result of the paucity of economic data on this industry is that the PPMI in Georgia has not been included in any of the 21 annual Farm Gate Value Reports (FGVRs) (CAED 2023). The FGVR provides revenue estimates for over 60 commodities in the agricultural industry in Georgia, while the USDA EC lists 36 commodities (CAED 2022, USDA 2023). Noncommodity revenue entries for the 2021 FGVR totaled approximately $1.09 billion despite those entities not being traditionally cultivated or animal husbandry industry sectors. If the PPMI industry is recognized by virtue of its regulatory relationship with the GDA, the total FGVR would increase by 5.67% or 6.73%. We propose the PPMI industry should be included in the FGVR, given the aforementioned precedent of including “nontraditional” revenues, because of that industry’s 50+ year close working relationship with, and regulatory oversight, by the GDA. The inclusion of the PPMI in agricultural economic statistics would draw attention to an overlooked partner for funding urban entomology programs within land grant institutions (Rust et al. 2023).

Our economic analysis of the PPMI in Georgia has illuminated aspects of the industry, including consistent growth throughout the 21st century (Figs. 1 and 2). More importantly, this work provides a template that anyone can follow to furnish transparent, verifiable, and comparable economic assessments at the state or national level with the caveat that between census years, estimates need to follow the forecast-based method. Lastly, the PPMI services stakeholders regardless of their proximity to traditional agriculture, yet we posit the close relationship the PPMI has with Agriculture oversight agencies—like the GDA—across the United States should be justification to include the PPMI when reporting annual agricultural economic revenue and contributions. Associating the economics of the PPMI with agriculture would highlight to land grant institutions, where urban entomology programs are administered, as well as policymakers the importance of building partnerships with a strong economic sector that services all citizens.

## Supplementary Material

toae029_suppl_Supplementary_Material_S1

toae029_suppl_Supplementary_Material_S2

toae029_suppl_Supplementary_Material_S3

toae029_suppl_Supplementary_Material_S4

toae029_suppl_Supplementary_Material_S5

toae029_suppl_Supplementary_Material_S6

toae029_suppl_Supplementary_Material_S7

toae029_suppl_Supplementary_Material_S8
