# Supplementary material for: Estimate of the revenue and economic contribution of the professional pest management industry in Georgia, United States
Source: J Econ Entomol. 2024 Feb 25;117(2):601–8. doi: 10.1093/jee/toae029 (PMC11011618; doi:10.1093/jee/toae029)
Supplement: toae029_suppl_Supplementary_Material_S1 [file toae029_suppl_supplementary_material_s1.docx]

**Pest Management Owner Questionnaire**

Thank you for taking the time to engage in this economic contribution survey for the pest management industry within Georgia. All survey responses are **ANONYMOUS** and **CANNOT** be traced back to respondents in any way.

**All questions are for the 2021 Fiscal Year.**

1. Select the **legal organization** of your business:

Sole Proprietorship  Partnership  LLC  C-Corporation  S-corporation

1. Year Established: ; Total number of offices/branches:
2. **List** states operated in: ; # of offices **outside** of GA: ___________
3. Percent of **Revenue** **generated in Georgia**: %
4. Permanent employees (**including yourself**):
   1. Owners:
   2. Certified Operators:
      1. Is an owner also the certified operator?  YES  NO
   3. Technicians:
   4. Office Staff:
5. Number of **temporary/seasonal** employees hired:
6. Number of **accounts/customers serviced** in 2021:
7. **Percent of accounts/customers** are in the following categories **(Must equal 100%)**:
   1. ______ % Residential (homes, apartments, etc.)
   2. ______ % Commercial (includes non-profits and government entities)
8. **Average number** **of visits** for each type of account/customer:
   1. ______ Residential (homes, apartments, etc.)
   2. ______ Commercial (includes non-profits and government entities)
9. **Revenue** for fiscal year **2021**: $
10. **Percent** of **revenue** generated by the following categories **(Must equal 100%)**:
    1. ______ % Household Pest Control (HPC)
    2. ______ % Wood-destroying Organism Control (WDO)
    3. ______ % Fumigation (FUM)
    4. ______ % Other:
11. **Expenses** for fiscal year **2021**: $
12. What percent of your business’s expenses were to the following categories?

(**Must equal 100%**)

- 1. ______% Payroll (wages, benefits, bonuses, etc.)
  2. ______% Vehicle Expenses (leasing, fuel, tires, maintenance/repairs, licensing)
  3. ______% Equipment and Pesticides
  4. ______% Property/Office Space (rent, maintenance, utilities)
  5. ______% Insurance
  6. ______% Subcontracting
  7. ______% General Administration (office supplies, computers, professional services)
  8. ______% Other

1. Have you received an offer to **sell** your company?

YES  NO

- 3
- 6
- 9
- 12
- 2
- 5
- 8
- 11
- 1
- 4
- 7
- 10

1.
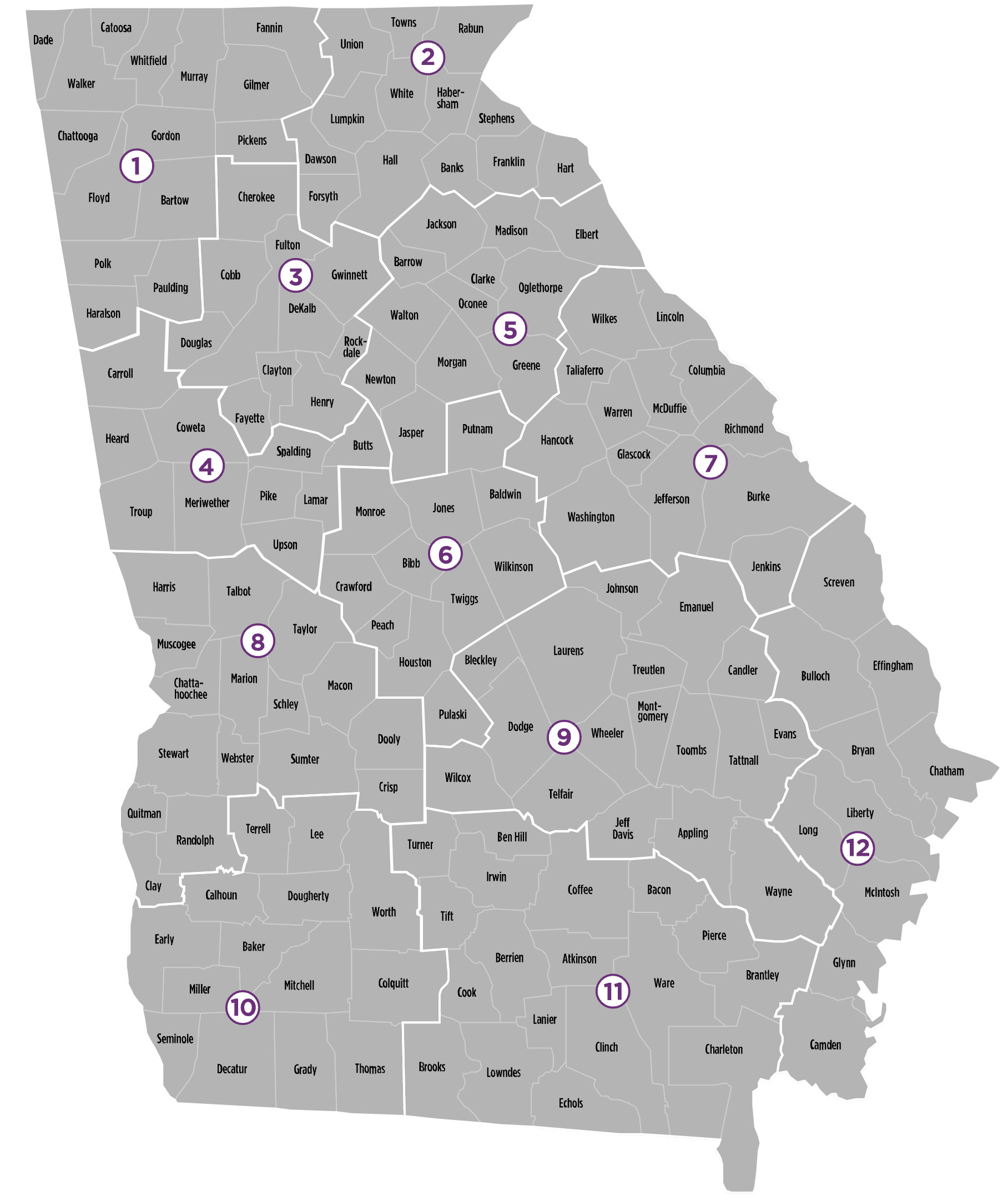
Please select the region(s) your business operates:
