# Supplementary material for: Estimate of the revenue and economic contribution of the professional pest management industry in Georgia, United States
Source: J Econ Entomol. 2024 Feb 25;117(2):601–8. doi: 10.1093/jee/toae029 (PMC11011618; doi:10.1093/jee/toae029)
Supplement: toae029_suppl_Supplementary_Material_S2 [file toae029_suppl_supplementary_material_s2.docx]

**Pest Management Employee Questionnaire**

Thank you for taking the time to engage in this economic contribution survey for the pest management industry within Georgia. All survey responses are **ANONYMOUS** and **CANNOT** be traced back to respondents in any way.

1. Select the **approximate size** of your company (administrative, office, technician).

Less than 5 employees  5-9 employees  10-19 employees  20-49 employees

50-99 employees  100-249 employees  More than 250 employees

1. Job title/position:
2. Average **number of days** worked out of 7-day week:
3. Average amount of **hours worked per week**:
4. Percent of time servicing the following categories (**Must equal 100%**):

a.______% Household Pest Control (HPC)

a. _____% Residential b.______% Wood-destroying Organism Control (WDO)

b. _____% Commercial c.______% Fumigation (FUM)

**100% (Total for column)** d.______% Other: _____________________________

**100% (Total for column)**

1. Number of accounts **serviced per workday**:  Not Applicable
2. Average time spent **servicing one account** (**minutes**):  Not Applicable
3. Select your **compensation method** and enter **compensation amount**:

Hourly $ hourly

Salary $ annually

Commission %

1. **Years** of pest management experience:
2. Which of the following **benefits** do you receive?

Health Insurance

Dental OR Vision Insurance

Retirement Savings Plan

Paid Vacation (**amount**: ___________ **per year**)

Sick Leave (**amount**: ___________ **per year**)

Bonuses

Take-Home Company Vehicle

Other:

1. Are you provided with the **appropriate resources** to complete your job efficiently? YES NO
2. If you checked **NO** on question **11**, please list the resources you are lacking:

1.
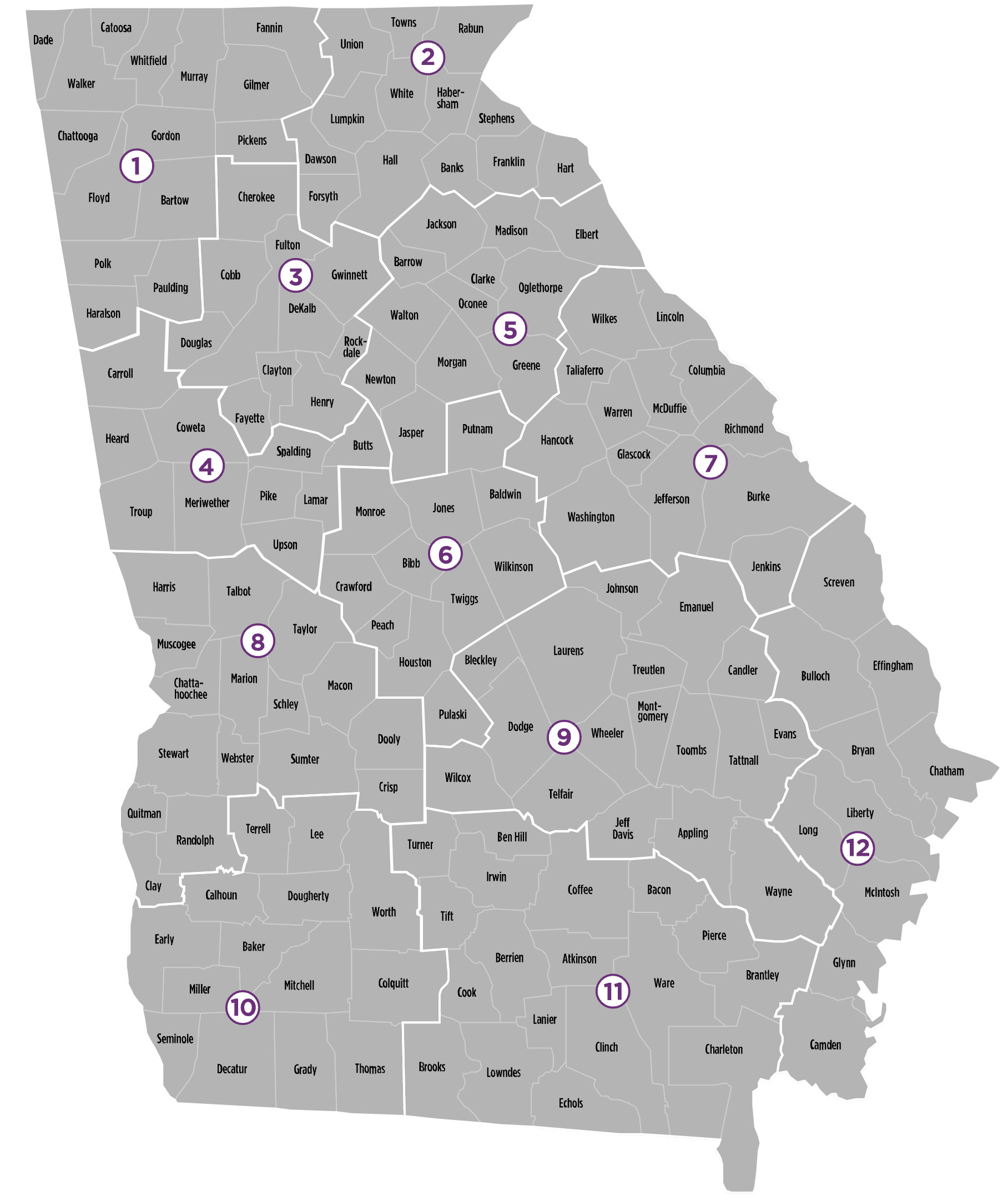
 Please select the **region(s)** you **service**:

- 3
- 6
- 9
- 12
- 2
- 5
- 8
- 11
- 1
- 4
- 7
- 10
