# Supplementary material for: Estimate of the revenue and economic contribution of the professional pest management industry in Georgia, United States
Source: J Econ Entomol. 2024 Feb 25;117(2):601–8. doi: 10.1093/jee/toae029 (PMC11011618; doi:10.1093/jee/toae029)

The process for acquiring 1997 employment data is shown, but the process for more recent data is identical by choosing the appropriate year in Step 7. Each step is illustrated as a screen shot of the webpage obtained by following the instructions at the top after the “Step” statement at the top of the page and selecting the choice identified within the red circle.

**Flow Chart to Access 1997 – Present**

**Quarterly Census of Employment and Wages**

**Step 1** – Go to <https://www.bls.gov>. The screen show below will be displayed.


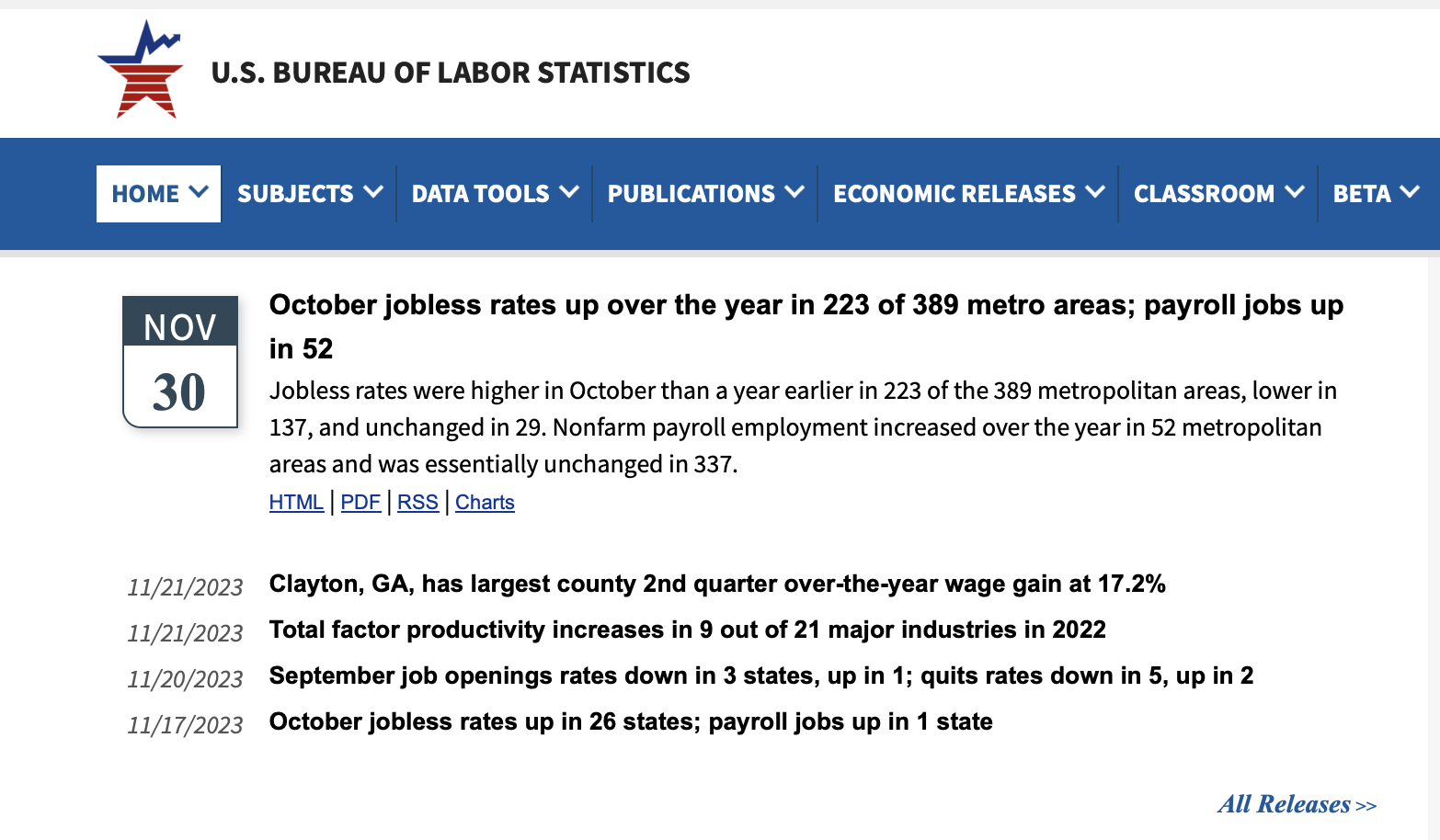


**Step 2** – Select “Subjects” in the sectional ribbon to display a drop-down menu.


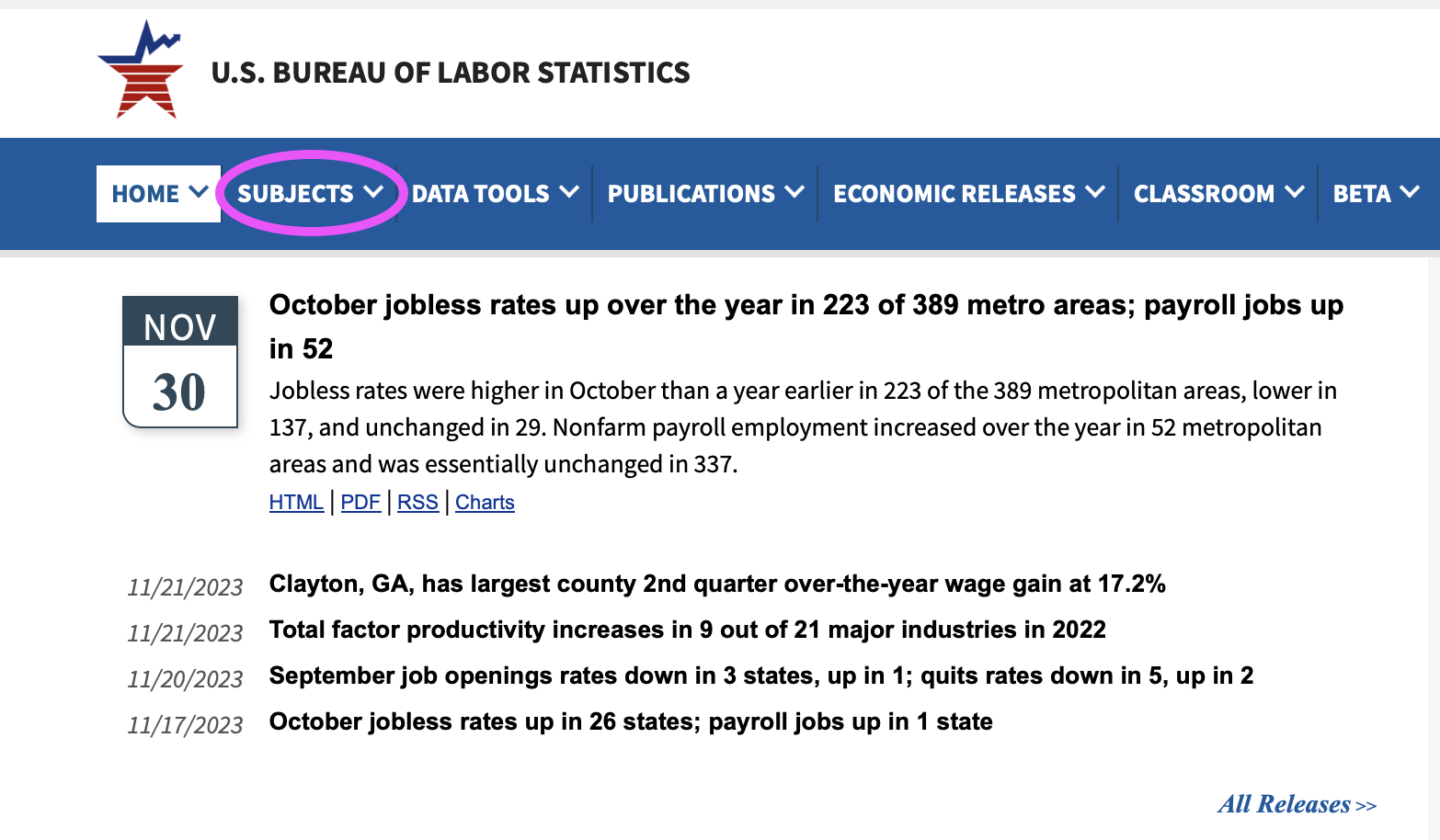


**Step 3** – Select “Employment” within the drop-down menu.


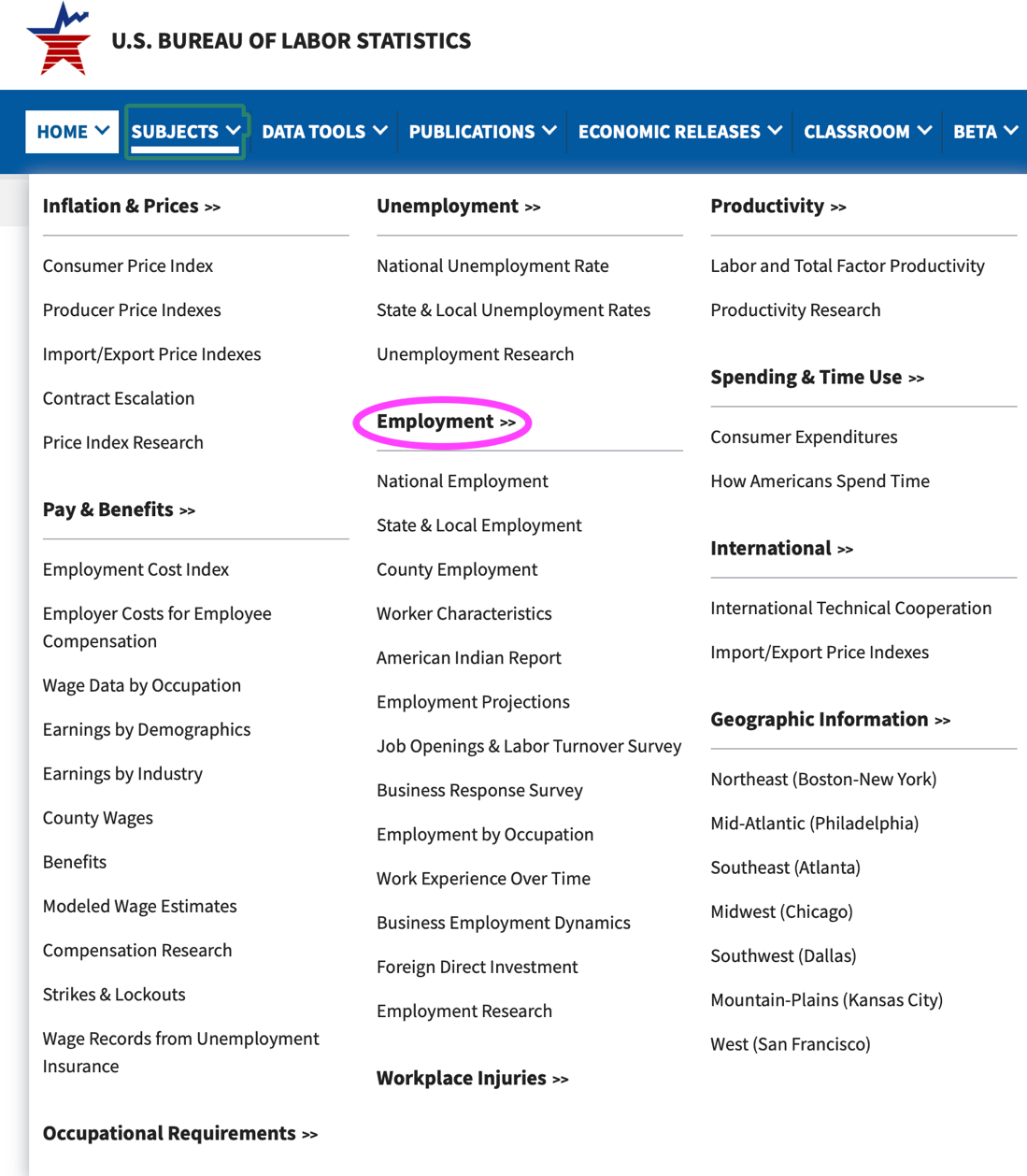


**Step 4** – Select “State and County Employment (Quarterly Census of Employment and Wages)” option.


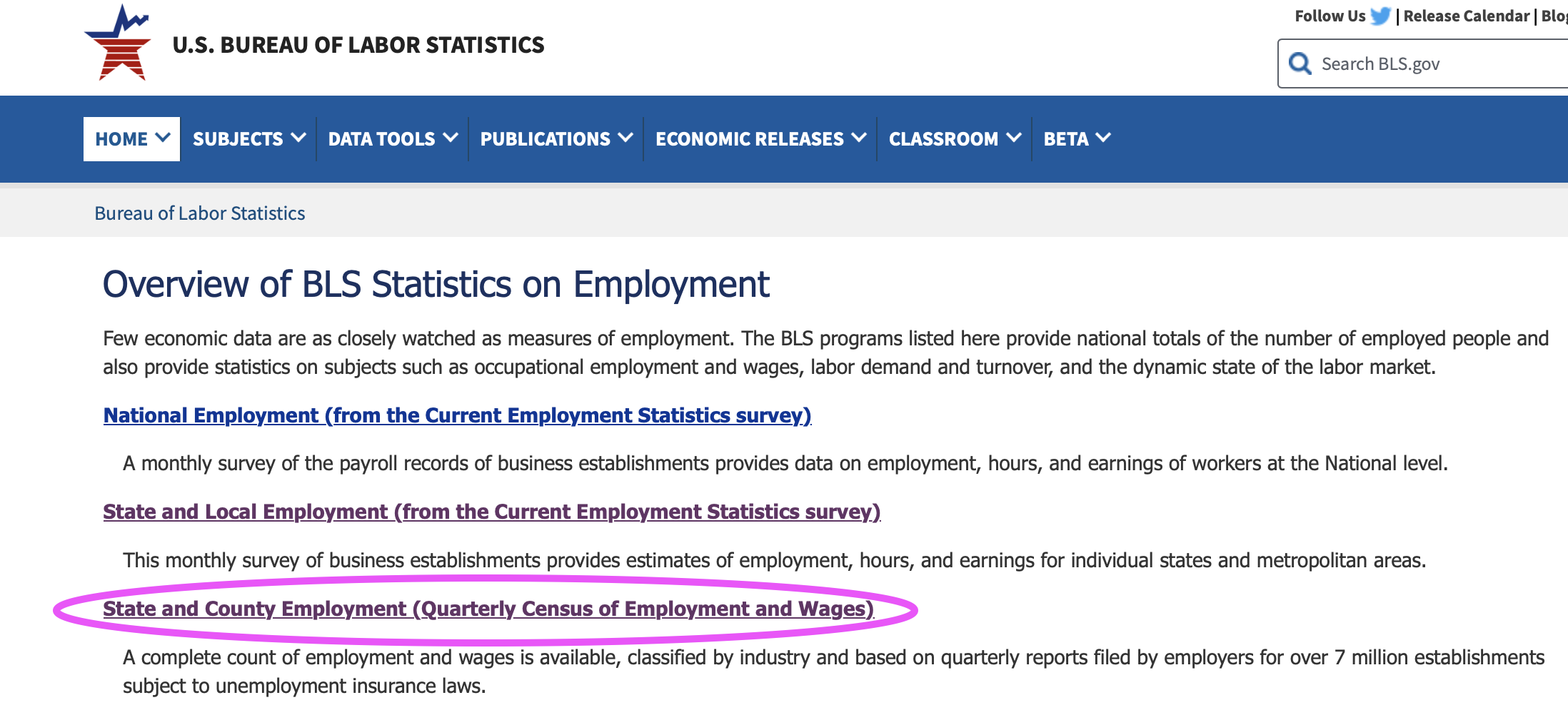


**Step 5** – Select “QCEW Data” to display the drop-down menu.


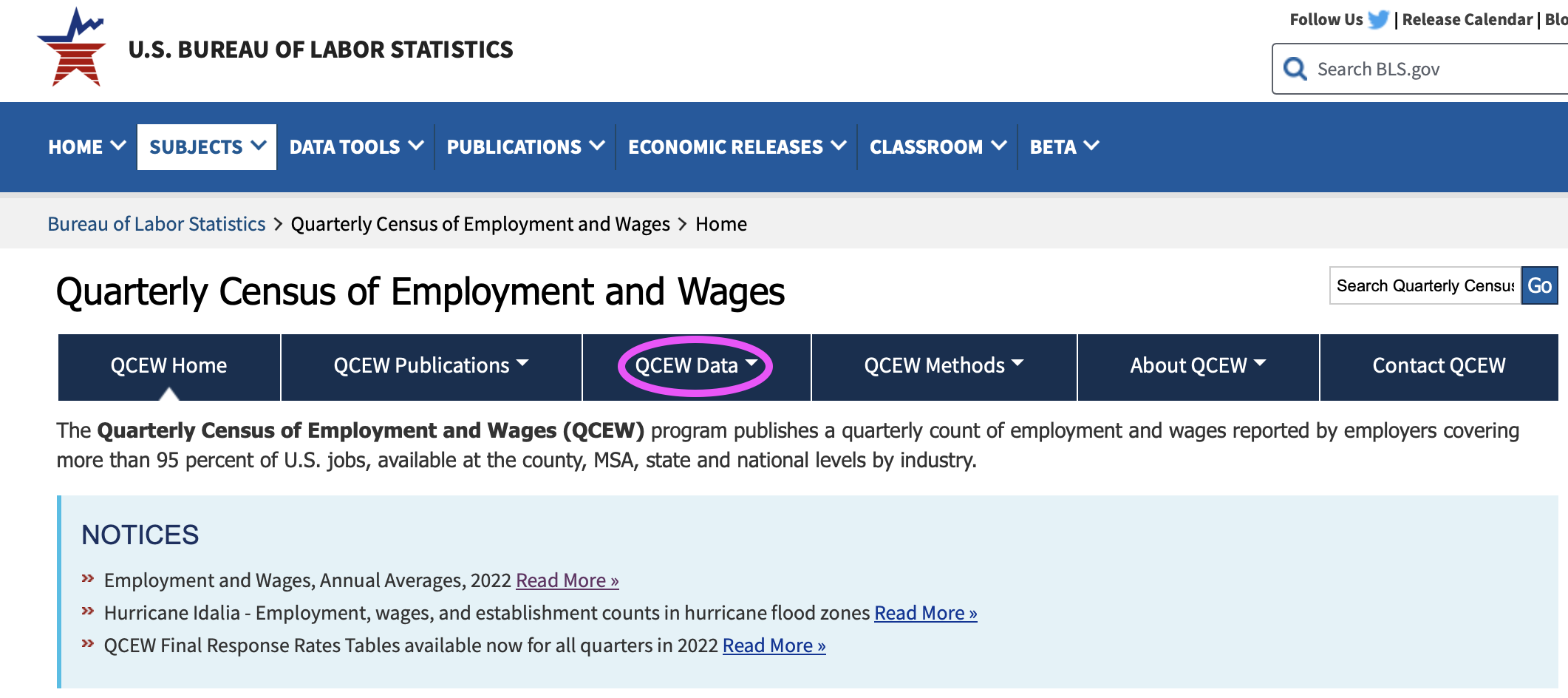


**Step 6** – Select “Downloadable Files.”


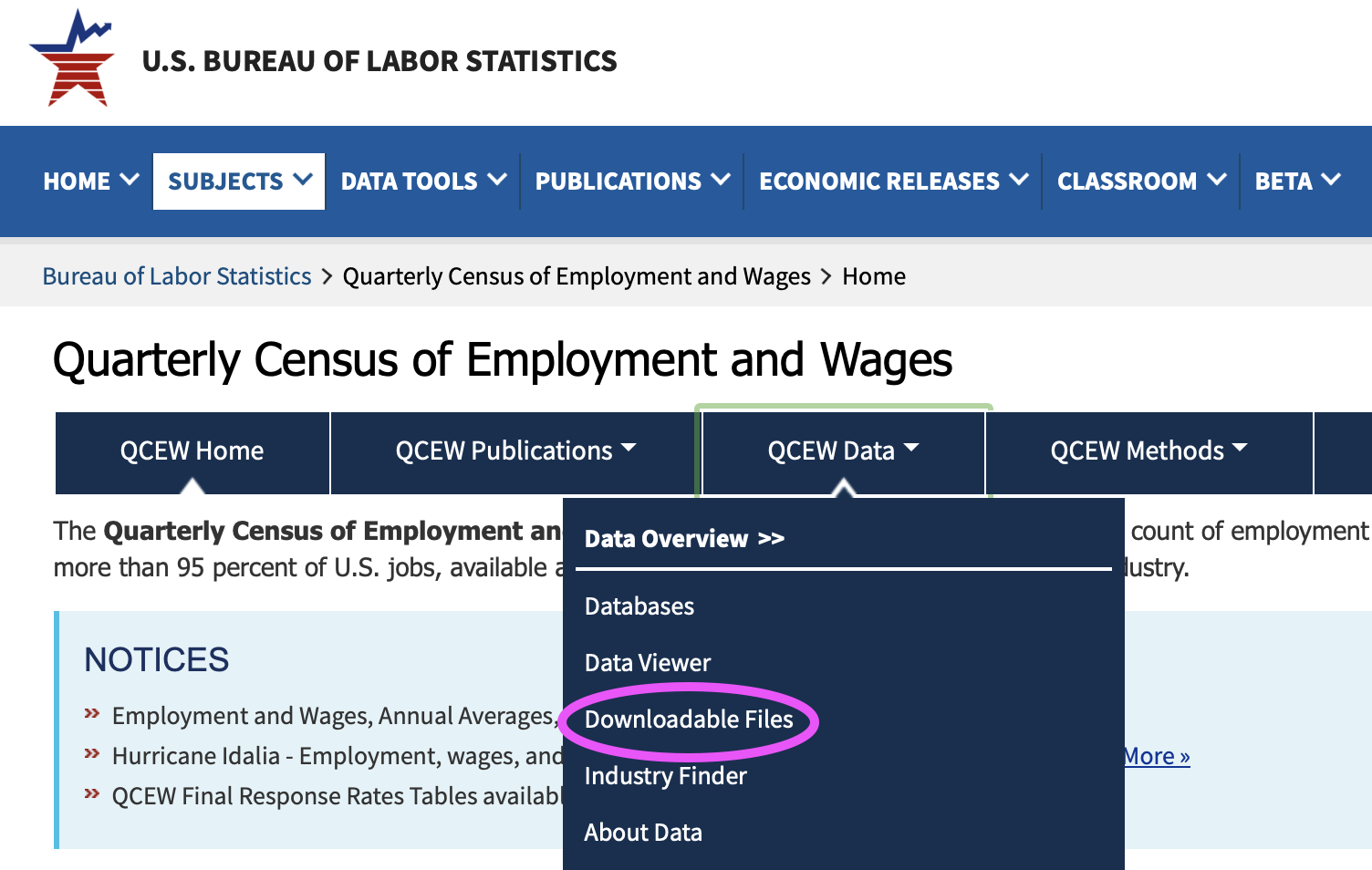


**Step 7** – Select any year from 1997 – to most recent data year under the “CSVs By Industry” column within the sub column “Annual Averages” to download.


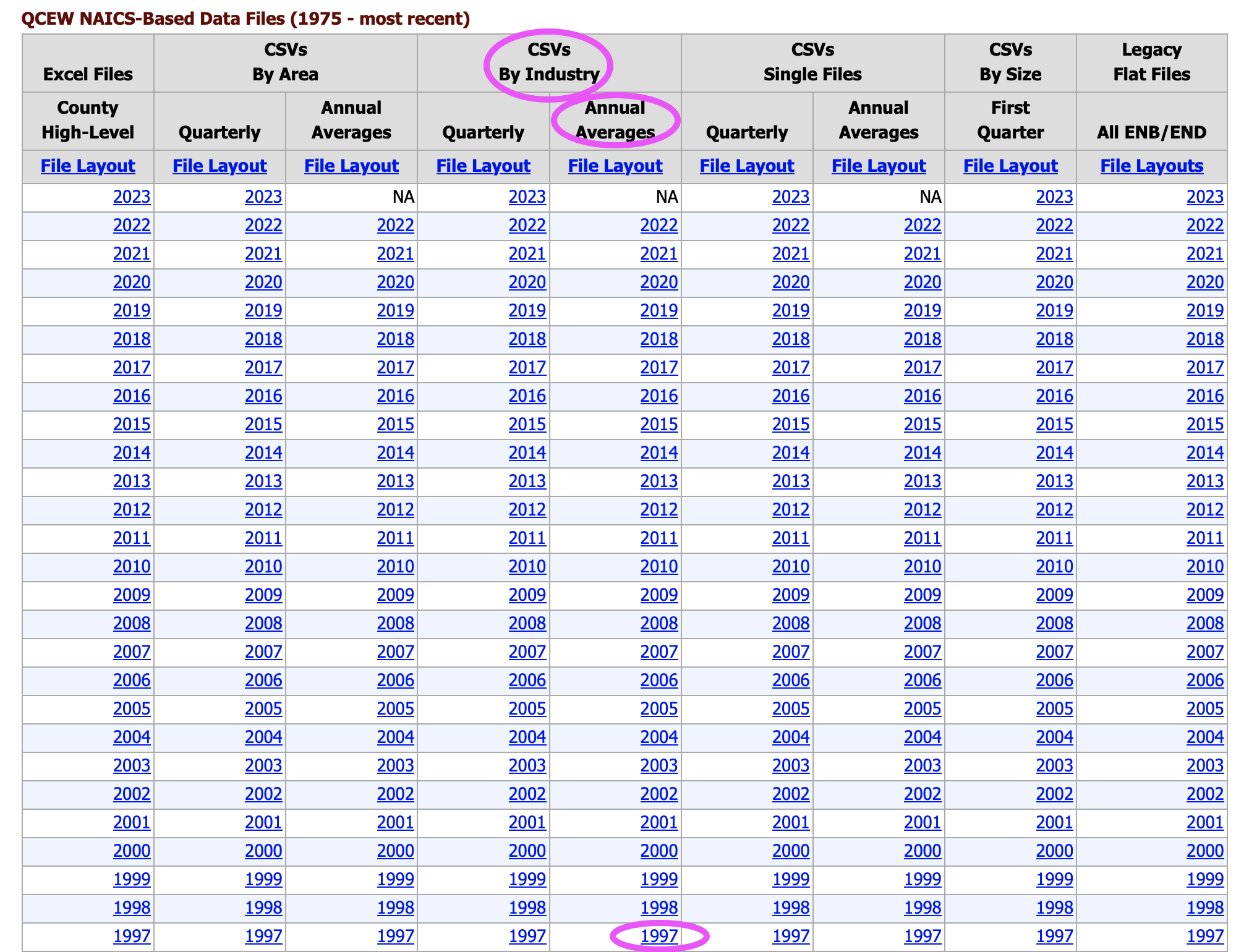


**Step 8** – The folder needs to be unzipped if downloaded in a .zip format. The folder contains employment data for every NAICS classification. Therefore, the professional pest management industry employment data is towards the end of the folder. Select the file that contains “561710 Exterminating and pest control services.”


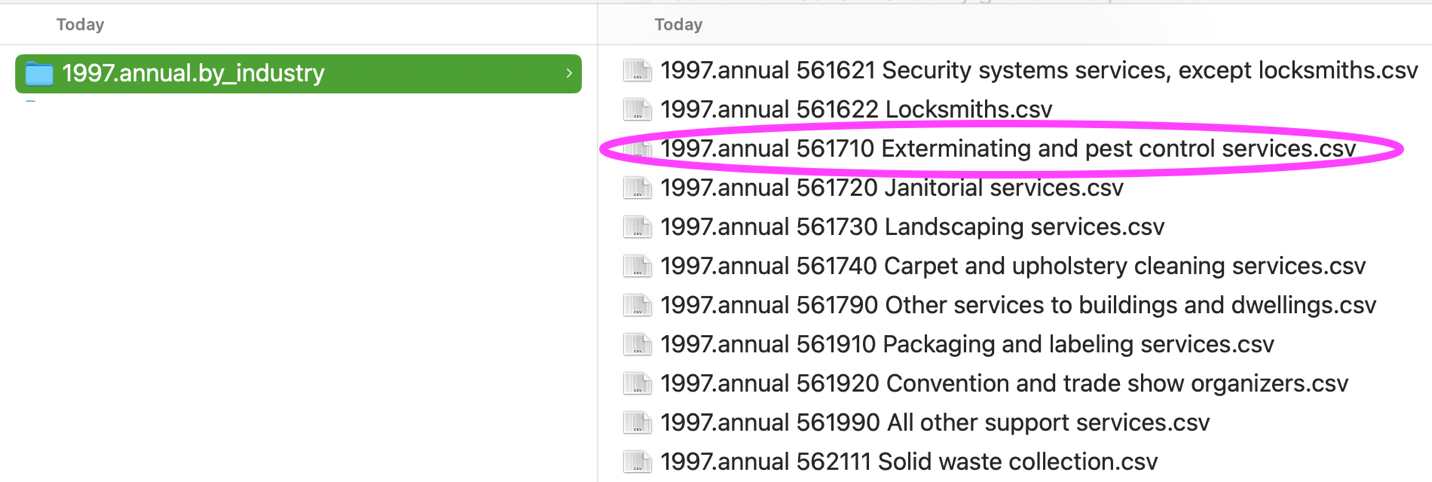

Supplement: toae029_suppl_Supplementary_Material_S3 [file toae029_suppl_supplementary_material_s3.docx]
