# Supplementary material for: Estimate of the revenue and economic contribution of the professional pest management industry in Georgia, United States
Source: J Econ Entomol. 2024 Feb 25;117(2):601–8. doi: 10.1093/jee/toae029 (PMC11011618; doi:10.1093/jee/toae029)

Acquiring historical revenue data process involves navigating changes in online reporting pathways. The process for acquiring 1997 & 2002 revenue data are similar. The revenue data for those years is identical by choosing the appropriate year (1997 & 2002) in step 3. Additionally, this flowchart depicts accessing Georgia PPMI revenue data; the process for all other states and national data are identical by specifying region of interest in step 7. The data for 1997 & 2002 were originally print publications, but they have been uploaded online as .pdf files with the Census Bureau’s integration of internet accessibility. Each step is illustrated as a screen shot of the webpage by following the instructions at the top after the “Step” statement at the top of the page and selecting the choice identified within the red circle.

**Flow Chart to Access 1997 & 2002**

**Economic Census Data**

**Step 1** – Go to <https://www.census.gov/programs-surveys/economic-census.html>. The screenshot below will be displayed.


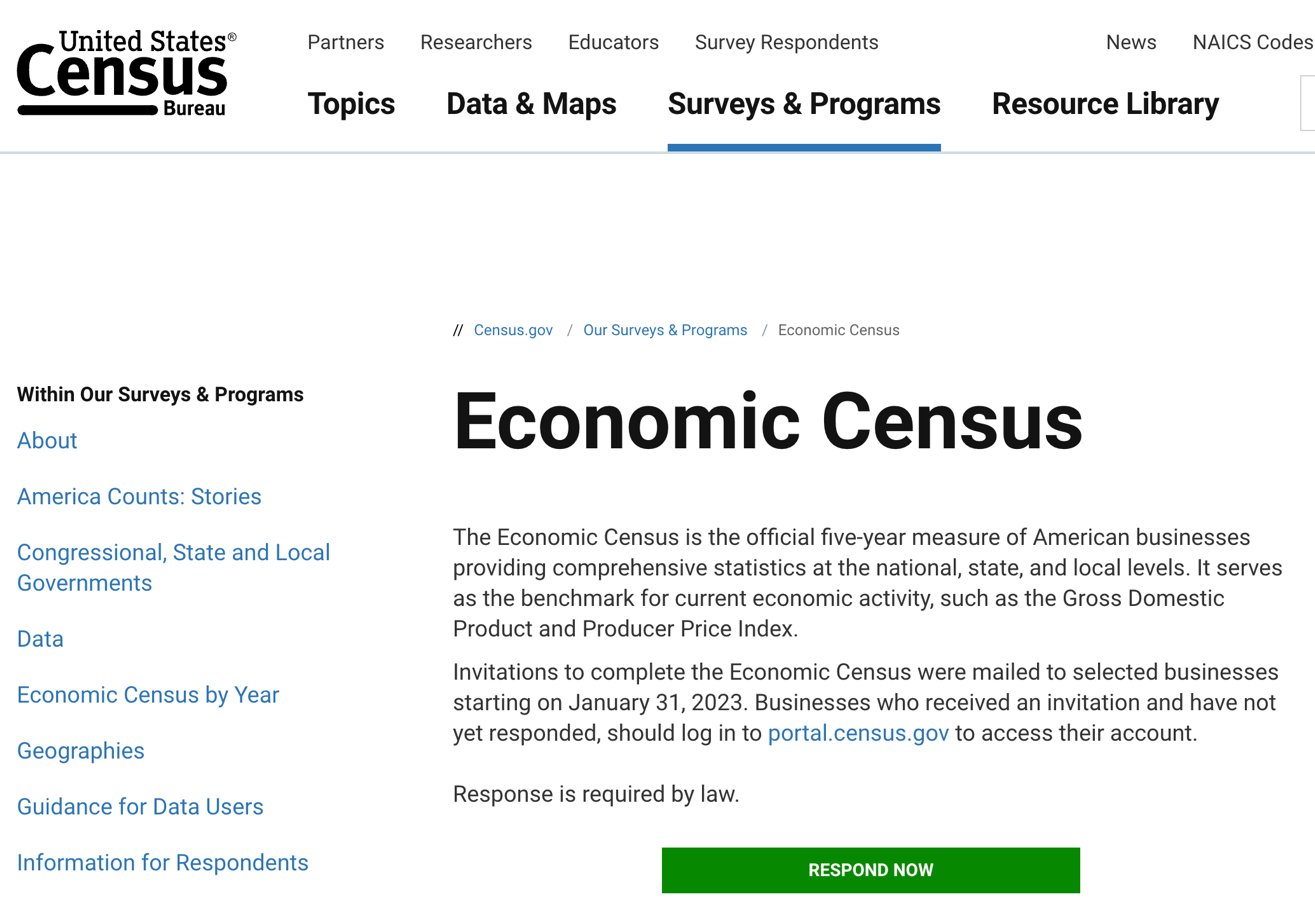


**Step 2** – Select “Economic Census by Year” in the left-hand sidebar.


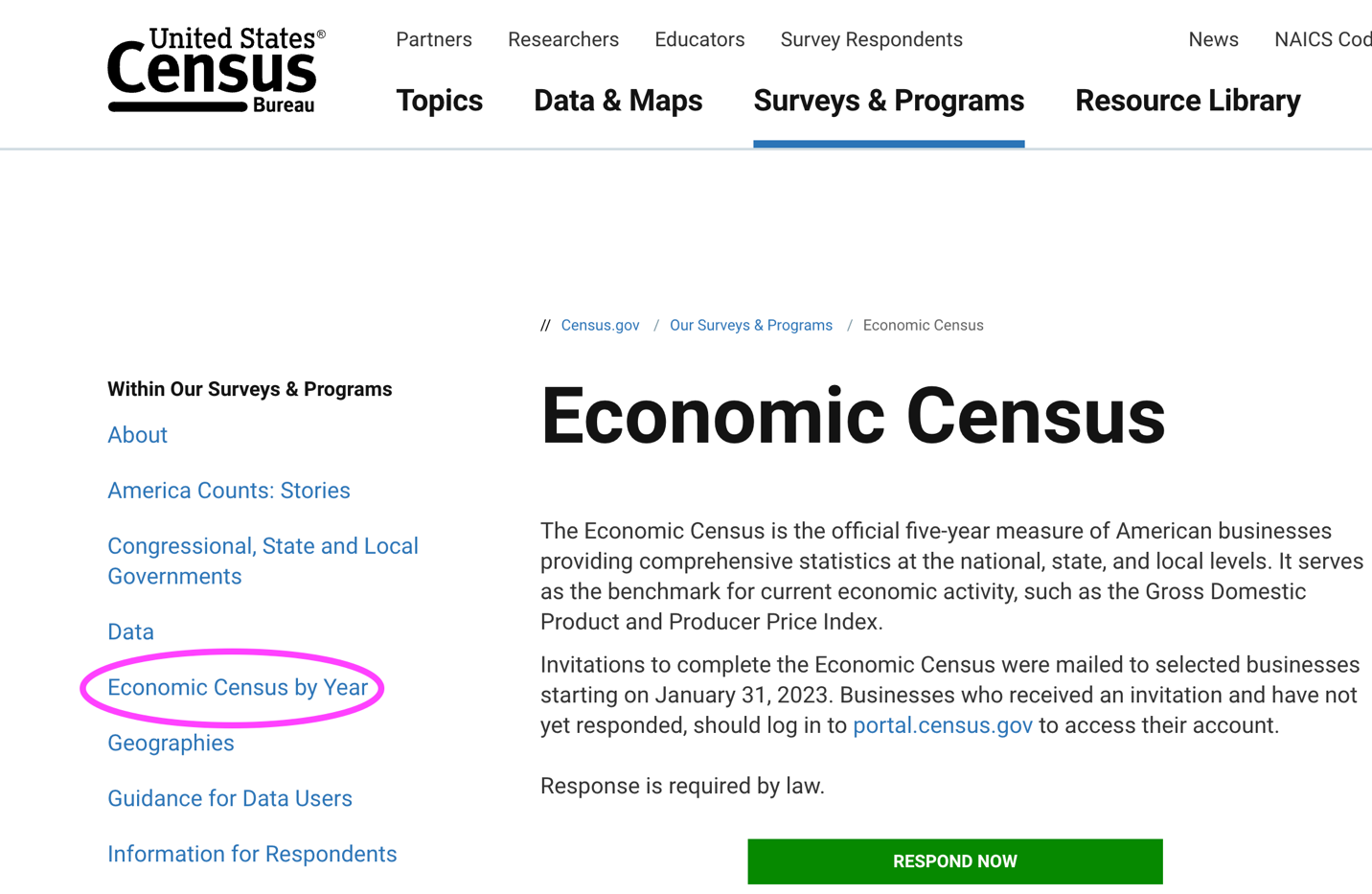


**Step 3** – Click on the drop-down menu labeled “More” to display more years available. Select either 1997.


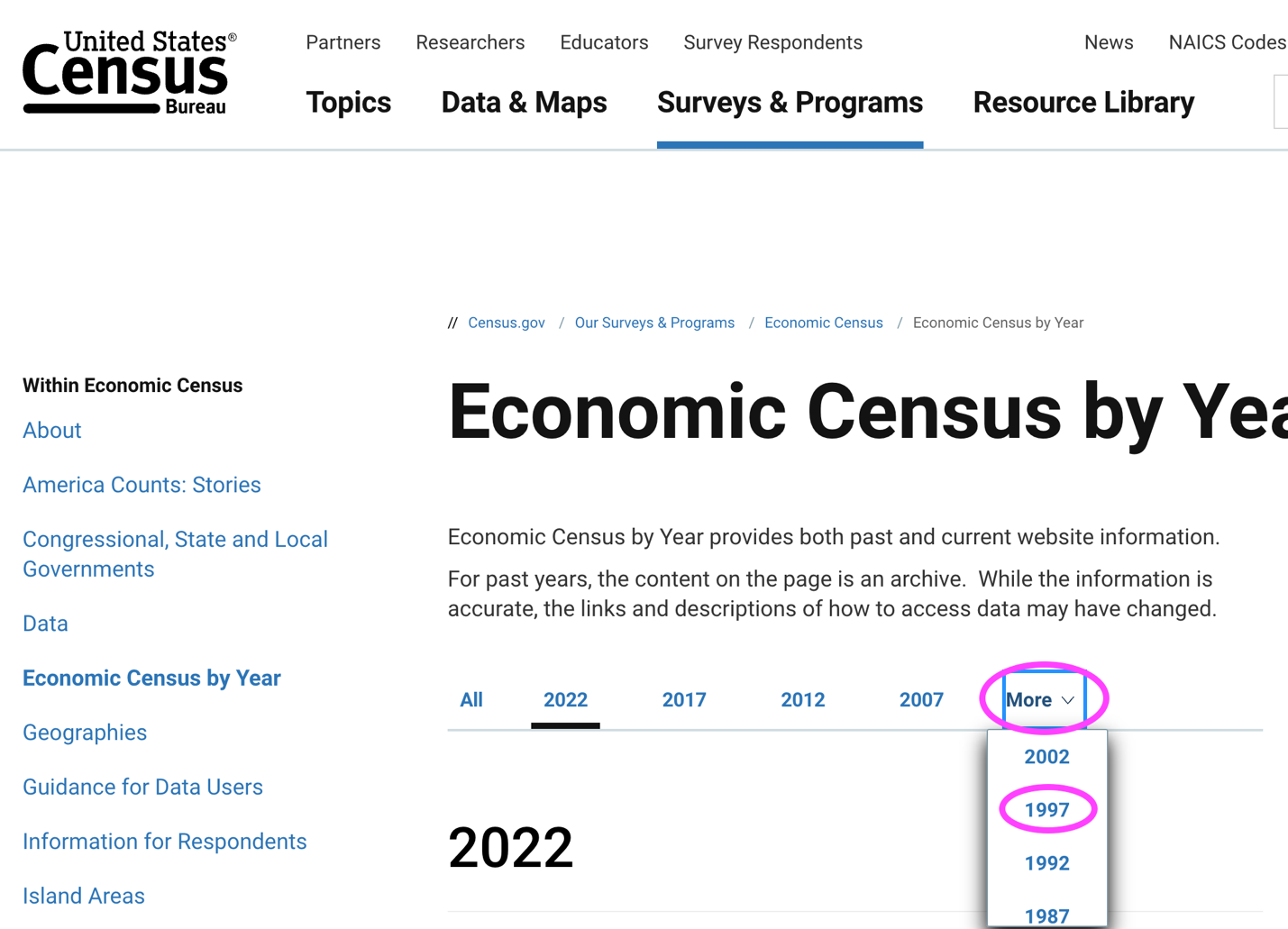


**Step 4** – Select the “1997 Economic Census Library” option.


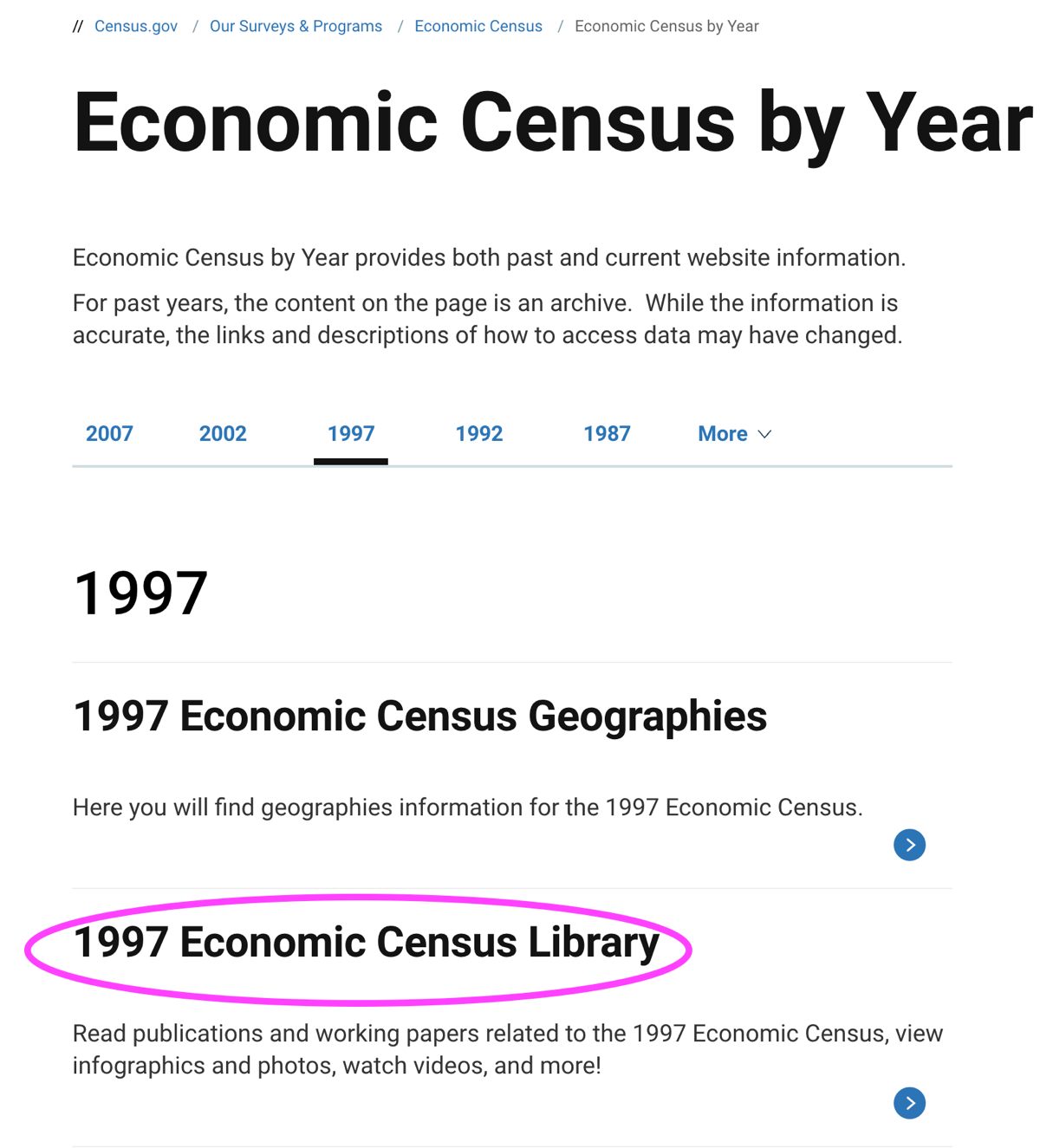


**Step 5** – Select “1997 Economic Census Publications.”


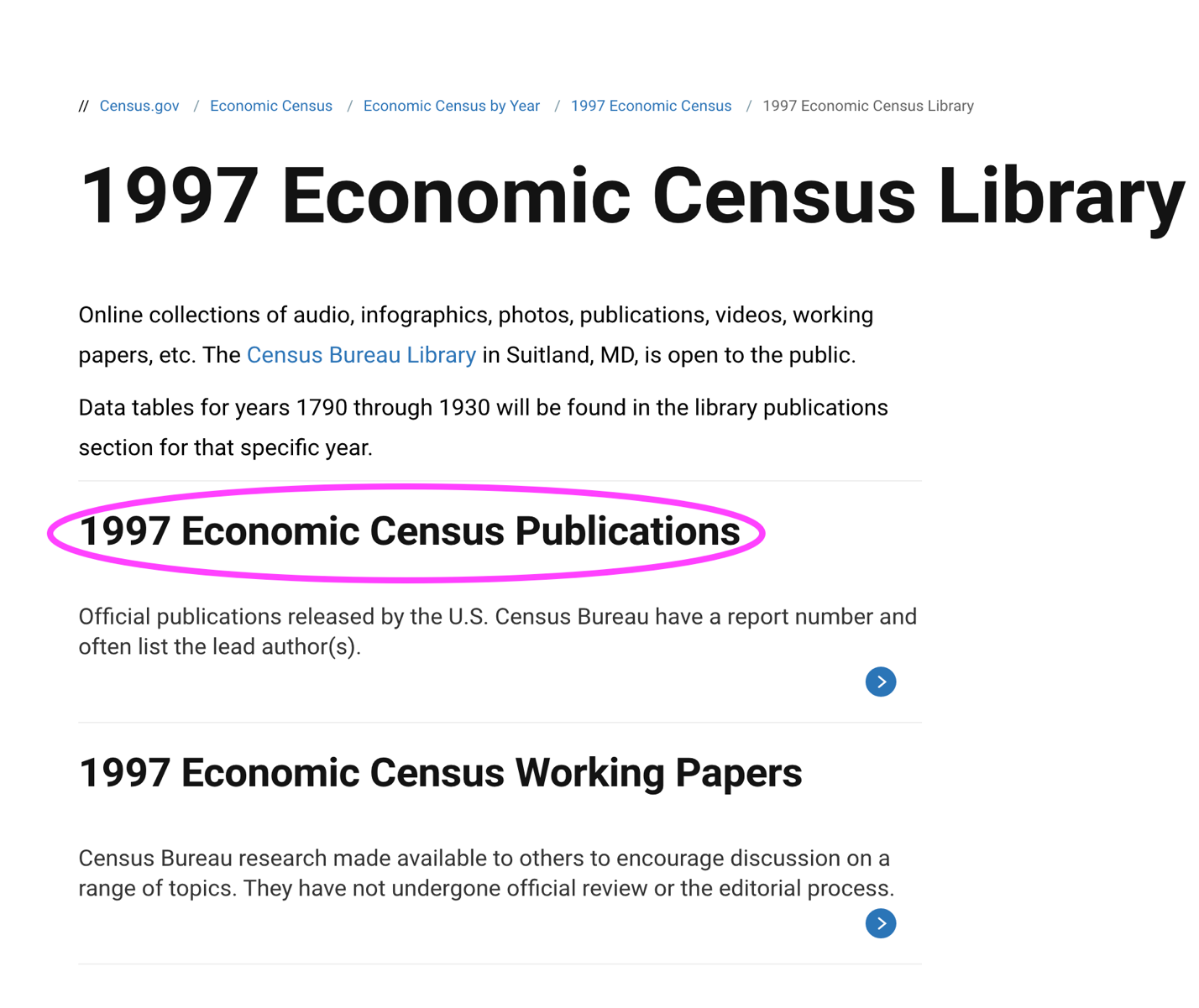


**Step 6** – Select the publication titled “Admin & Support & Waste Mgmt & Remediation Svcs (NAICS Sector 56).”


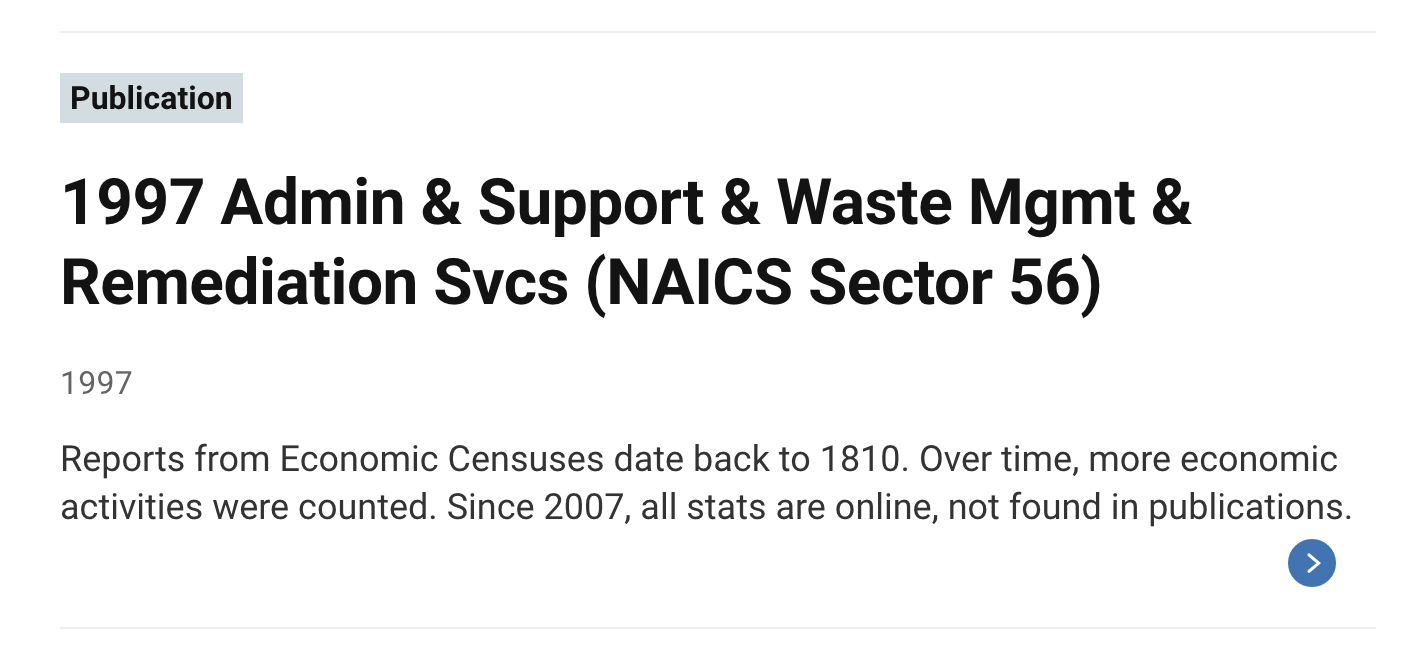


**Step 7** – Select “Georgia” within the “Select a State” dropdown menu. The publication automatically downloads in .pdf format.


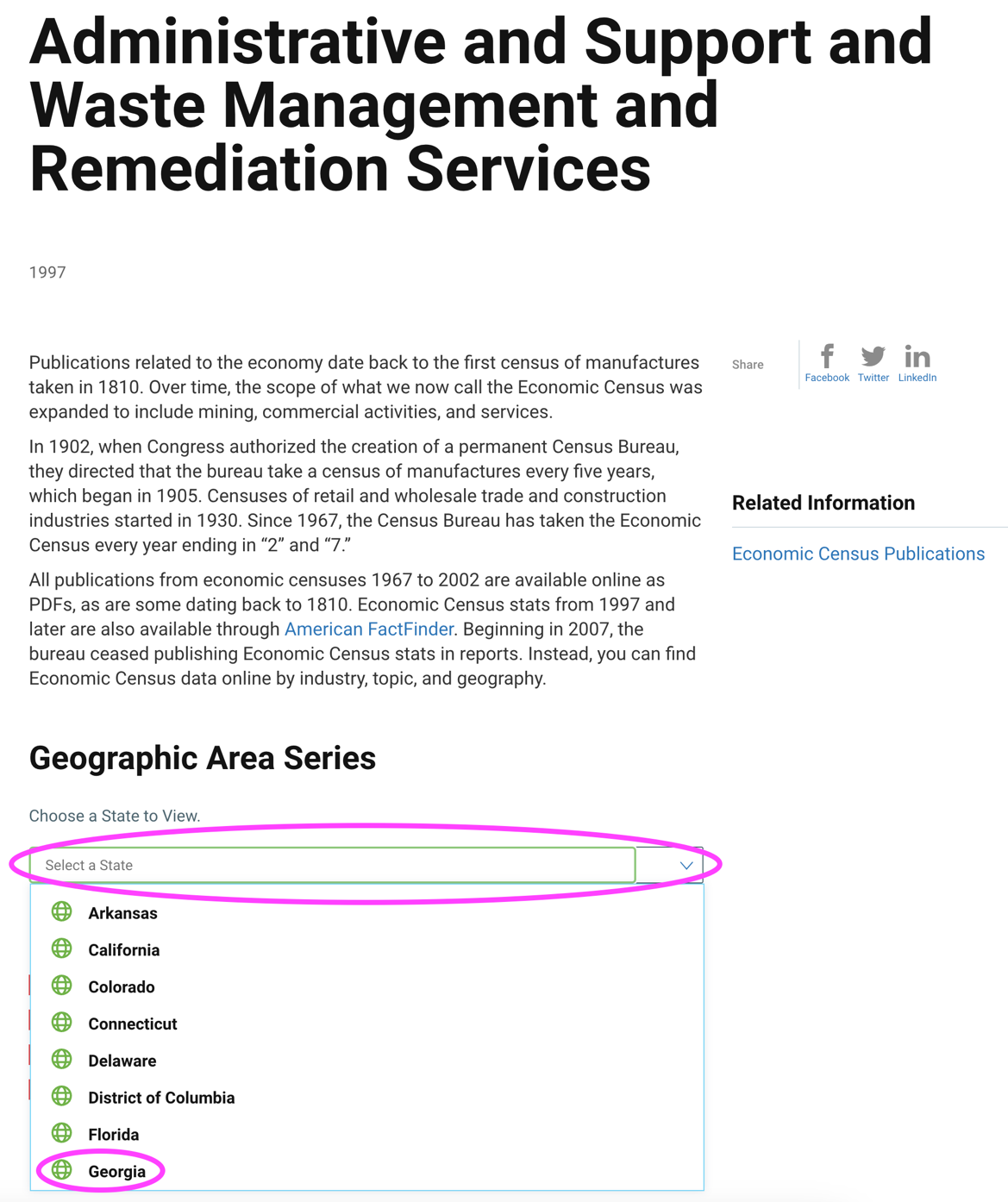

Supplement: toae029_suppl_Supplementary_Material_S4 [file toae029_suppl_supplementary_material_s4.docx]
