# Supplementary material for: Estimate of the revenue and economic contribution of the professional pest management industry in Georgia, United States
Source: J Econ Entomol. 2024 Feb 25;117(2):601–8. doi: 10.1093/jee/toae029 (PMC11011618; doi:10.1093/jee/toae029)

Acquiring historical revenue data process involves navigating changes in online reporting pathways. The process for acquiring 2007 revenue data is shown. This year of data is the least accessible because of changes made in the progression of internet accessibility within the Census Bureau. The .csv file selected in step 9 contains all 50 states and national revenue data for the PPMI. Each step is illustrated as a screen shot of the webpage obtained by following the instructions at the top after the “Step” statement at the top of the page and selecting the choice identified within the red circle.

**Flow Chart to Access 2007**

**Economic Census Data**

**Step 1** – Go to <https://www.census.gov/programs-surveys/economic-census.html>. The screen shot below will be displayed.


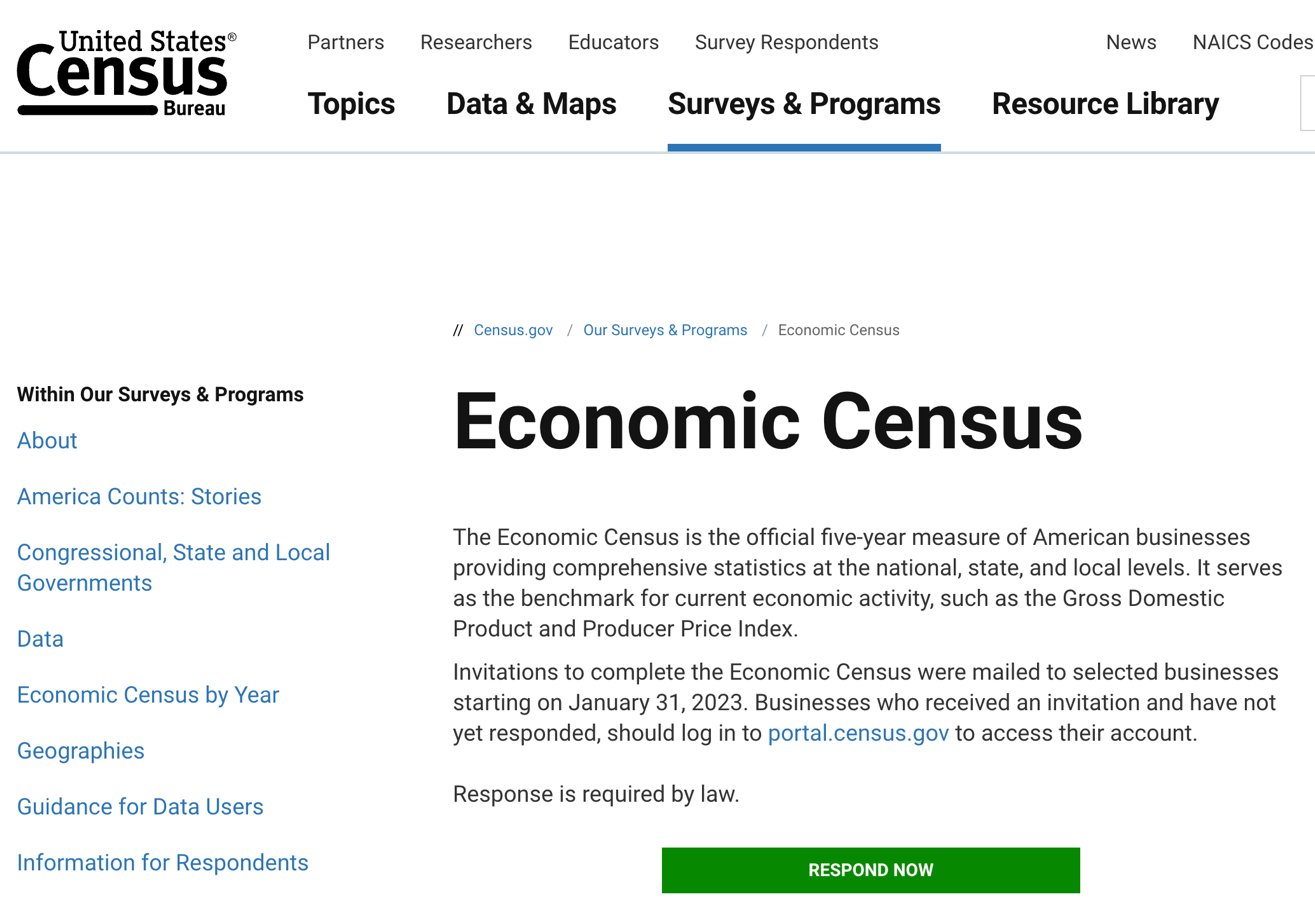


**Step 2** – Select “Economic Census by Year” in the left-hand sidebar.


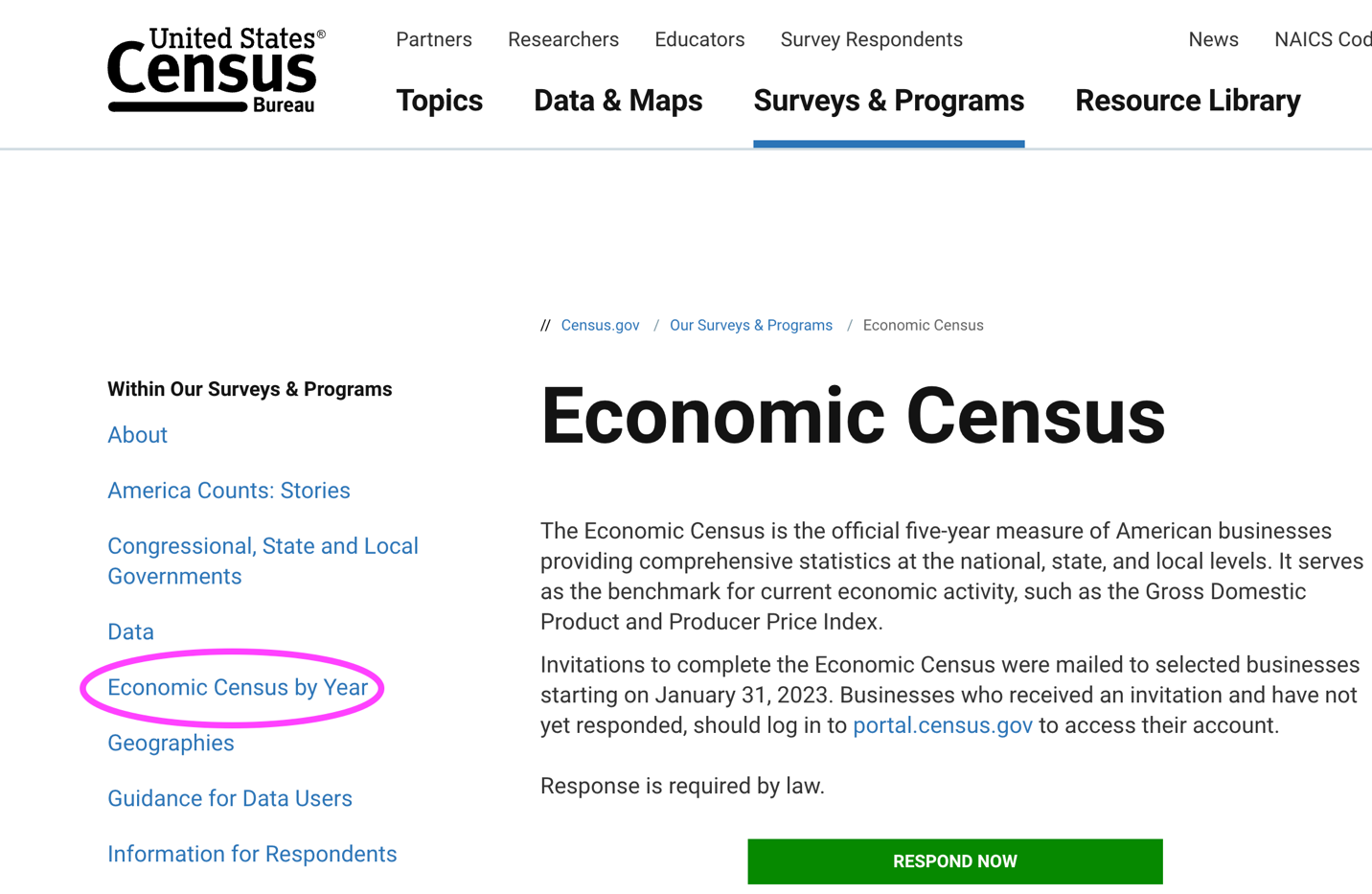


**Step 3** – Click on “2007” in the year ribbon.


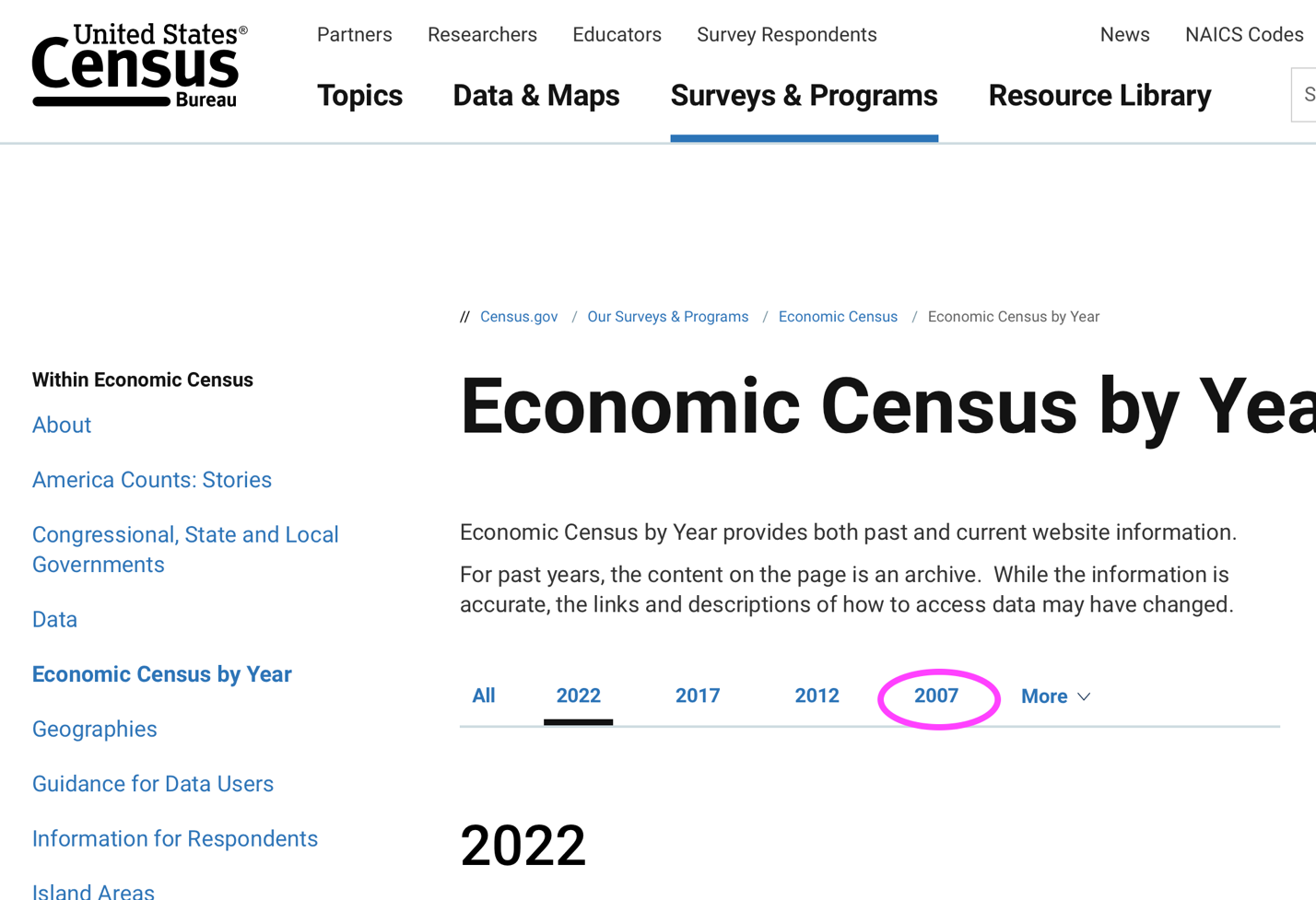


**Step 4** – Select the “2007 Economic Census Data” option.


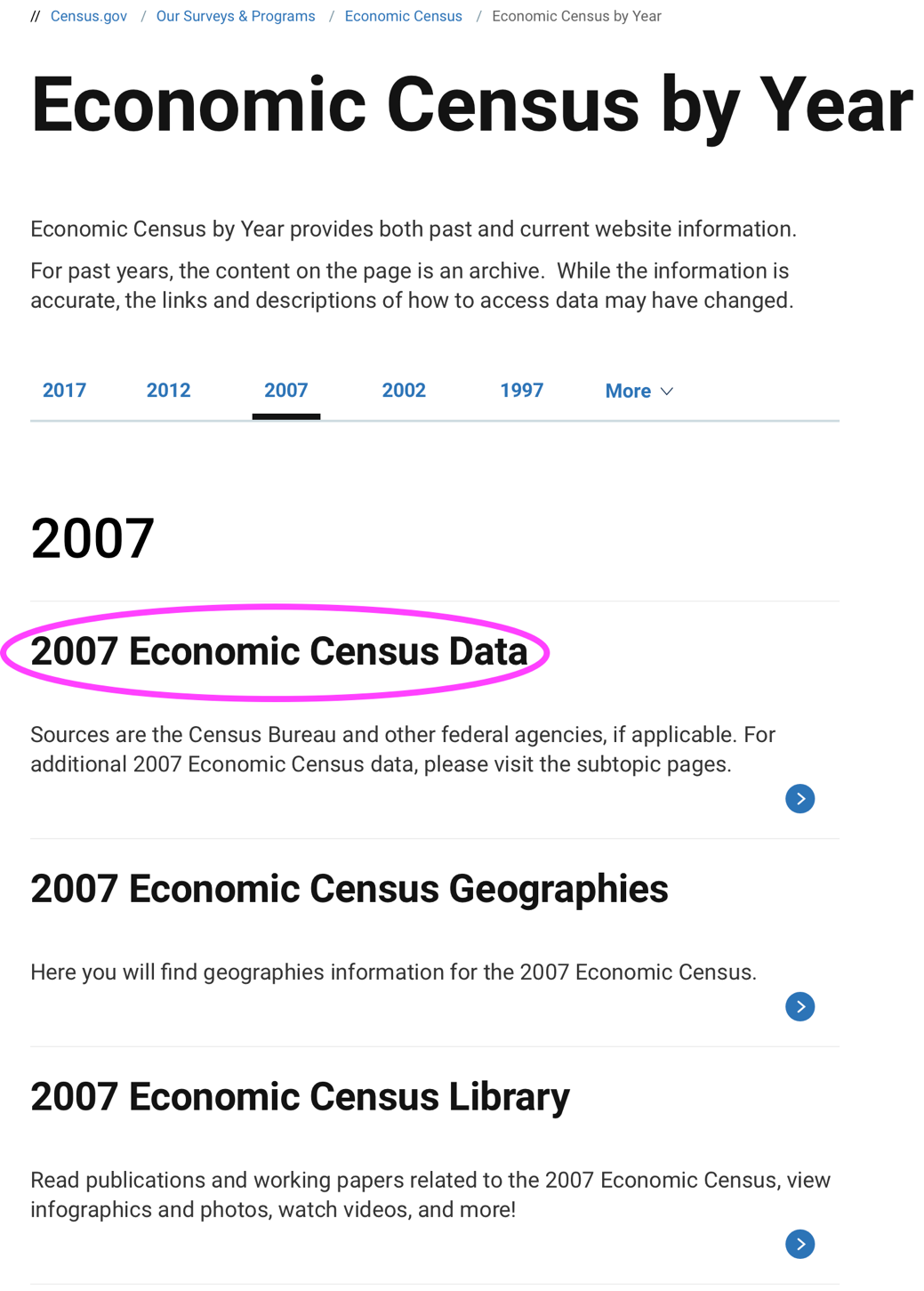


**Step 5** – Select “2007 Economic Census Data Tables.”


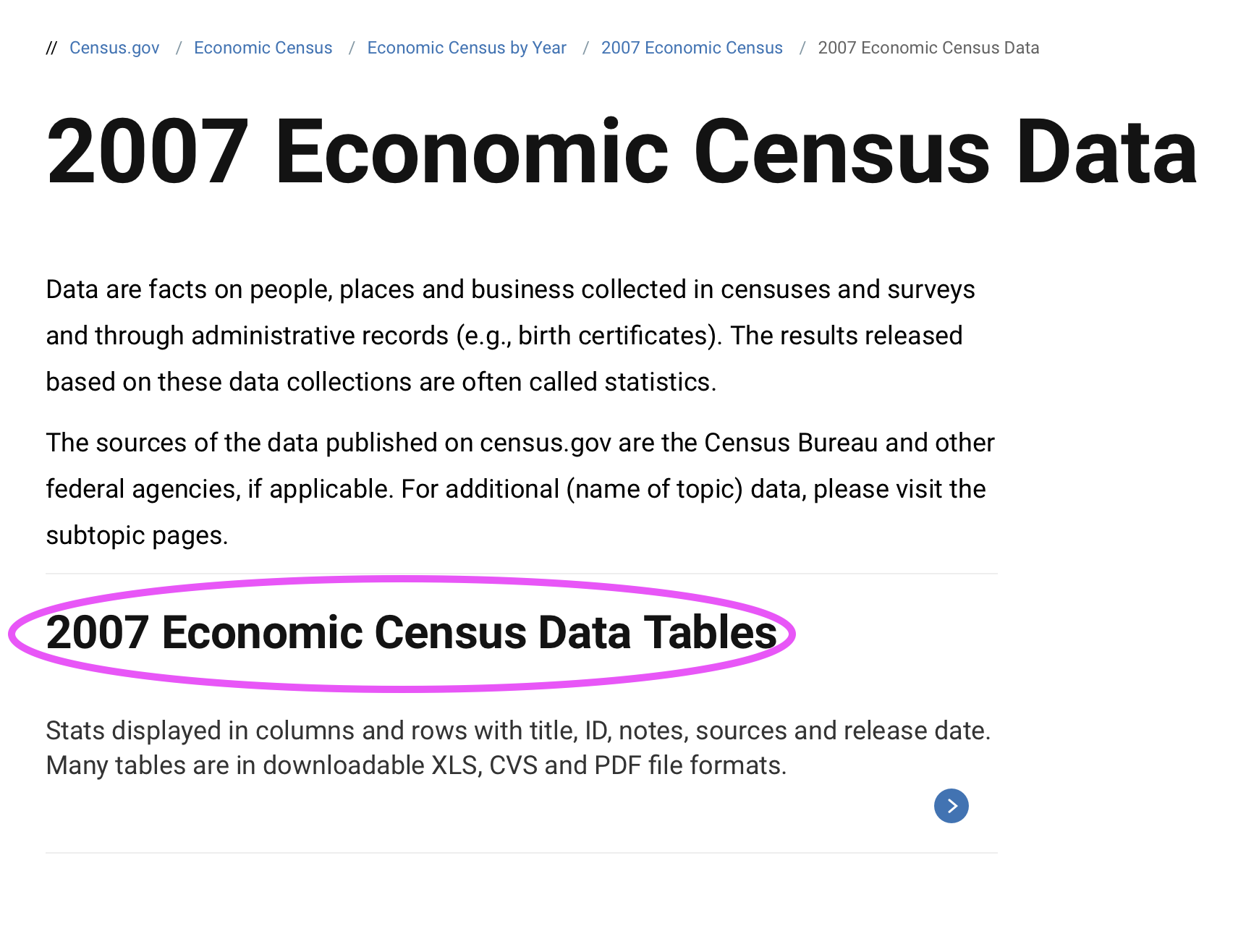


**Step 6** – Select the option titled “2007 NAICS Sector 56 - Admin & Support & Waste Mgmt & Remediation Svcs.”


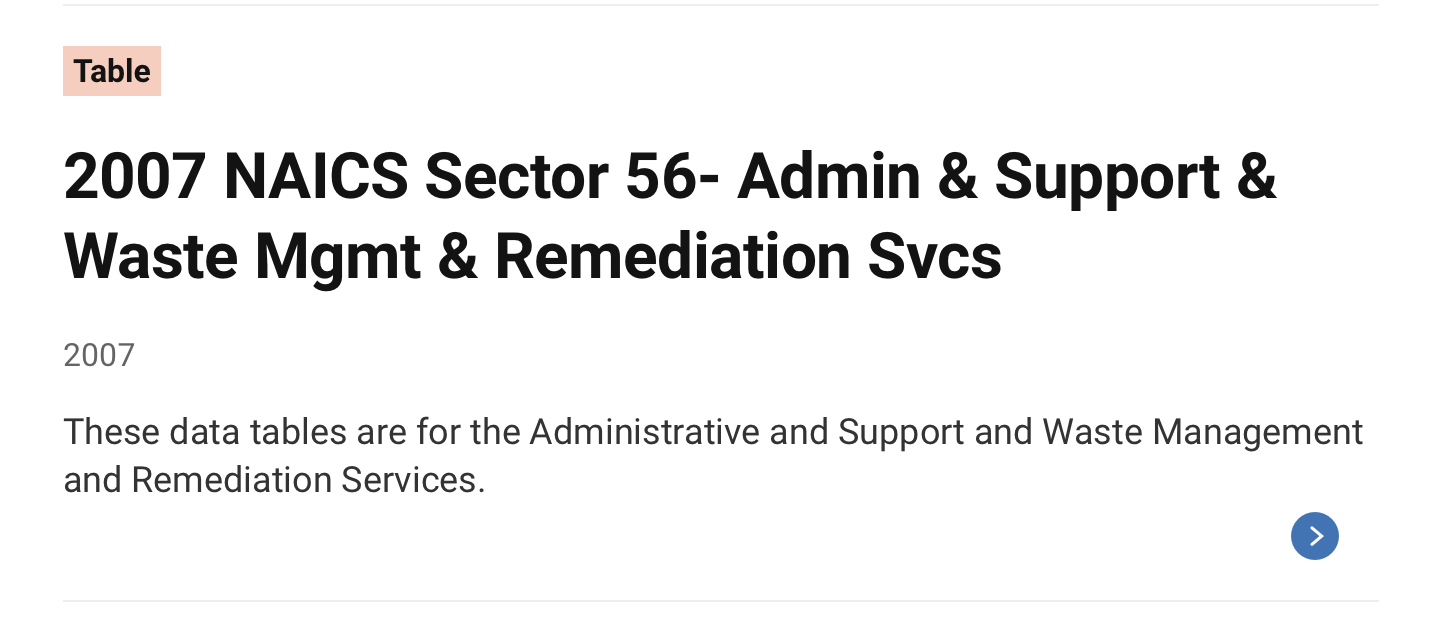


**Step 7** – Select “FTP Directory” within the section titled “Geographic Area Series.”


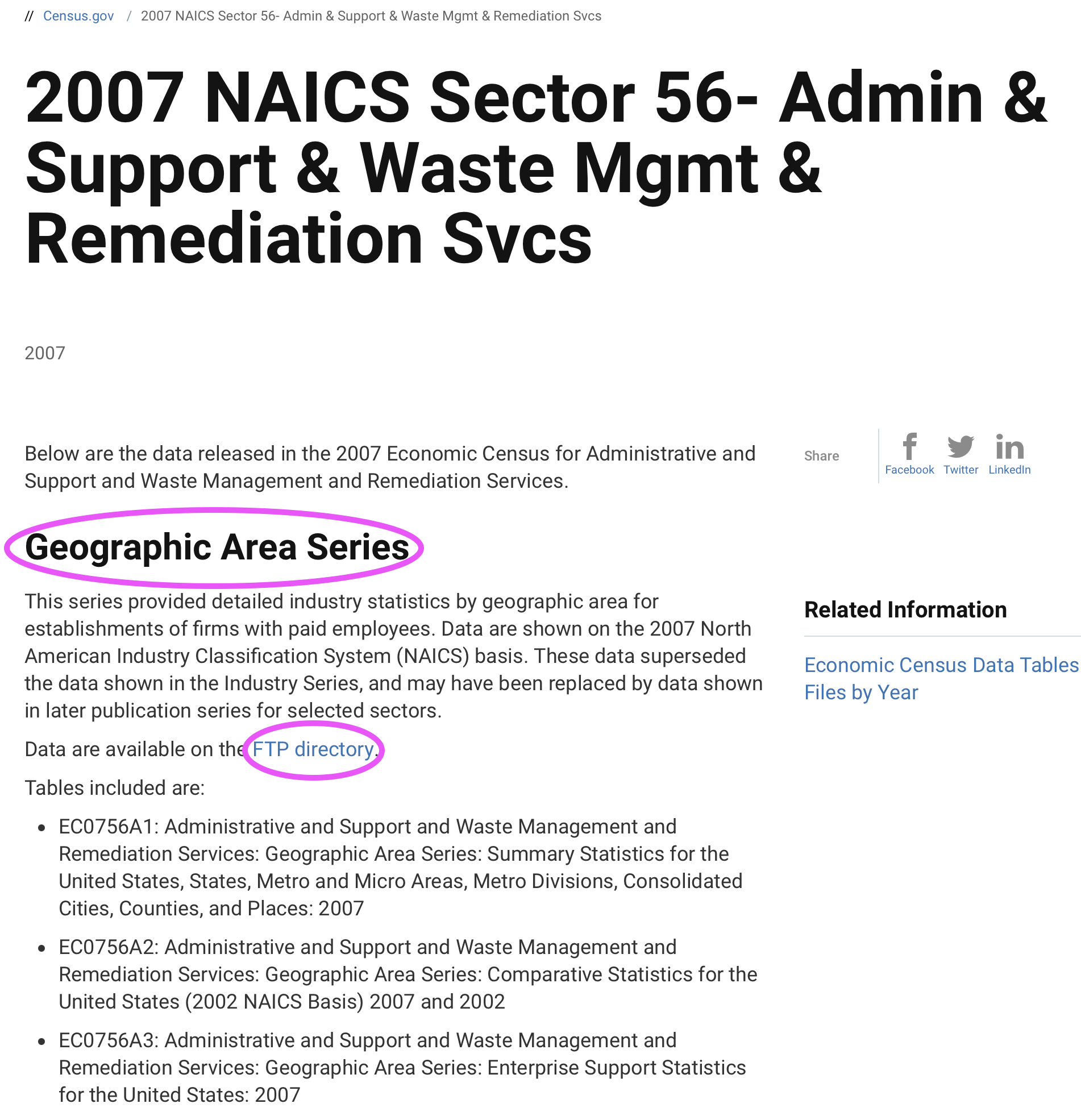


**Step 8** – Select the folder “2007_56_GAS_State.zip” to download.


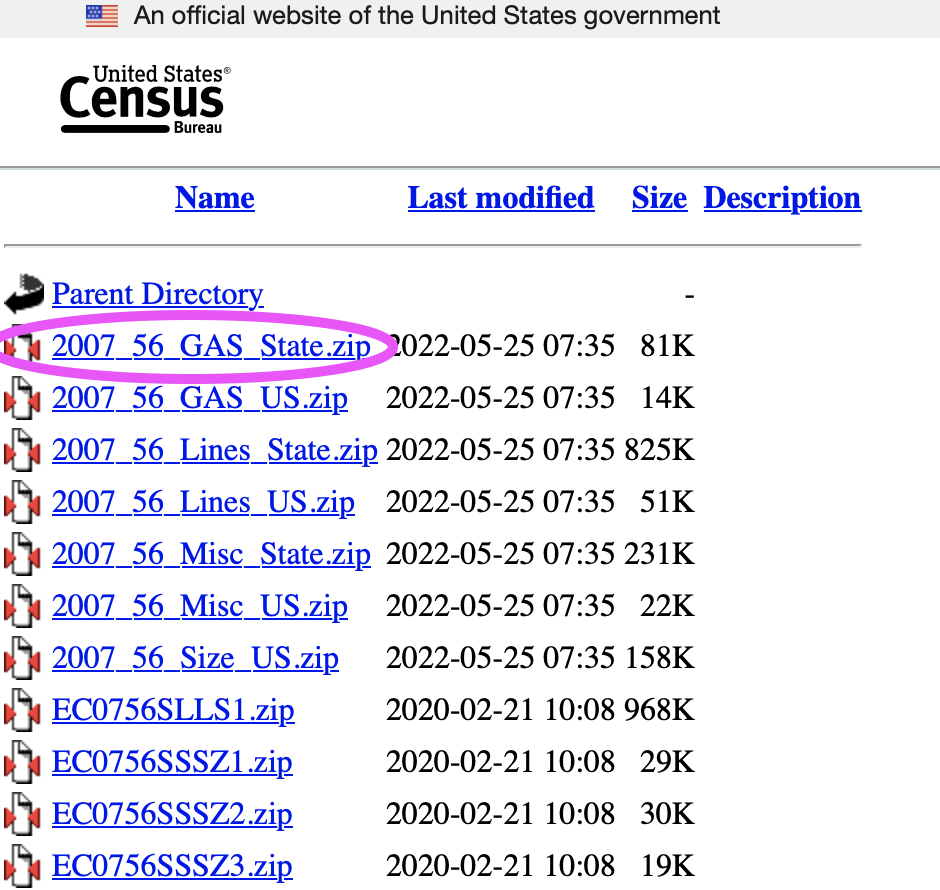


**Step 9** – Extract the .zip folder downloaded in the previous step. Select the file named “ECN_2007_US_56A1_with_ann.csv” to access both state and national data for 561710.


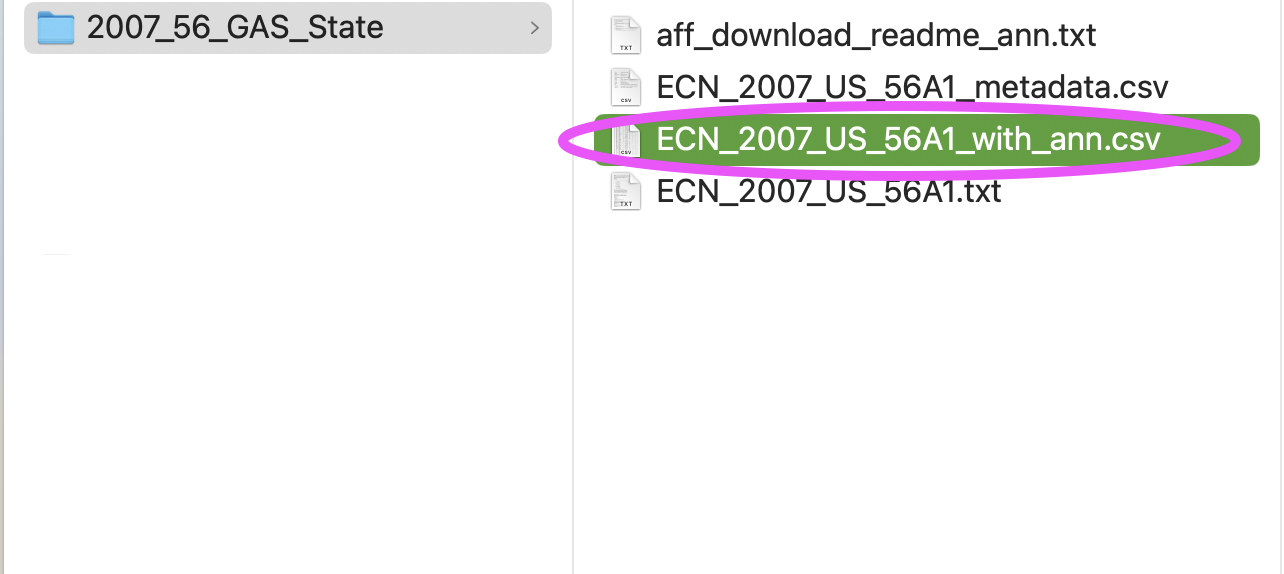

Supplement: toae029_suppl_Supplementary_Material_S5 [file toae029_suppl_supplementary_material_s5.docx]
