# Supplementary material for: Estimate of the revenue and economic contribution of the professional pest management industry in Georgia, United States
Source: J Econ Entomol. 2024 Feb 25;117(2):601–8. doi: 10.1093/jee/toae029 (PMC11011618; doi:10.1093/jee/toae029)

Acquiring historical revenue data process involves navigating changes in online reporting pathways. The process for acquiring 2012 and 2017 revenue data is shown. This flowchart depicts accessing Georgia PPMI revenue data; the process for all other states and national data are identical by specifying region of interest in step 3. These two Economic Census years are the first available in the Census Bureau’s interactive database website, and subsequent years should be available through this site contingent upon the Bureau’s discretion. Each step is illustrated as a screen shot of the webpage by following the instructions at the top after the “Step” statement at the top of the page and selecting the choice identified within the red circle.

**Flow Chart to Access 2012 & 2017**

**Economic Census Data**

**Step 1** – Go to <https://data.census.gov>. The screenshot below will be displayed.


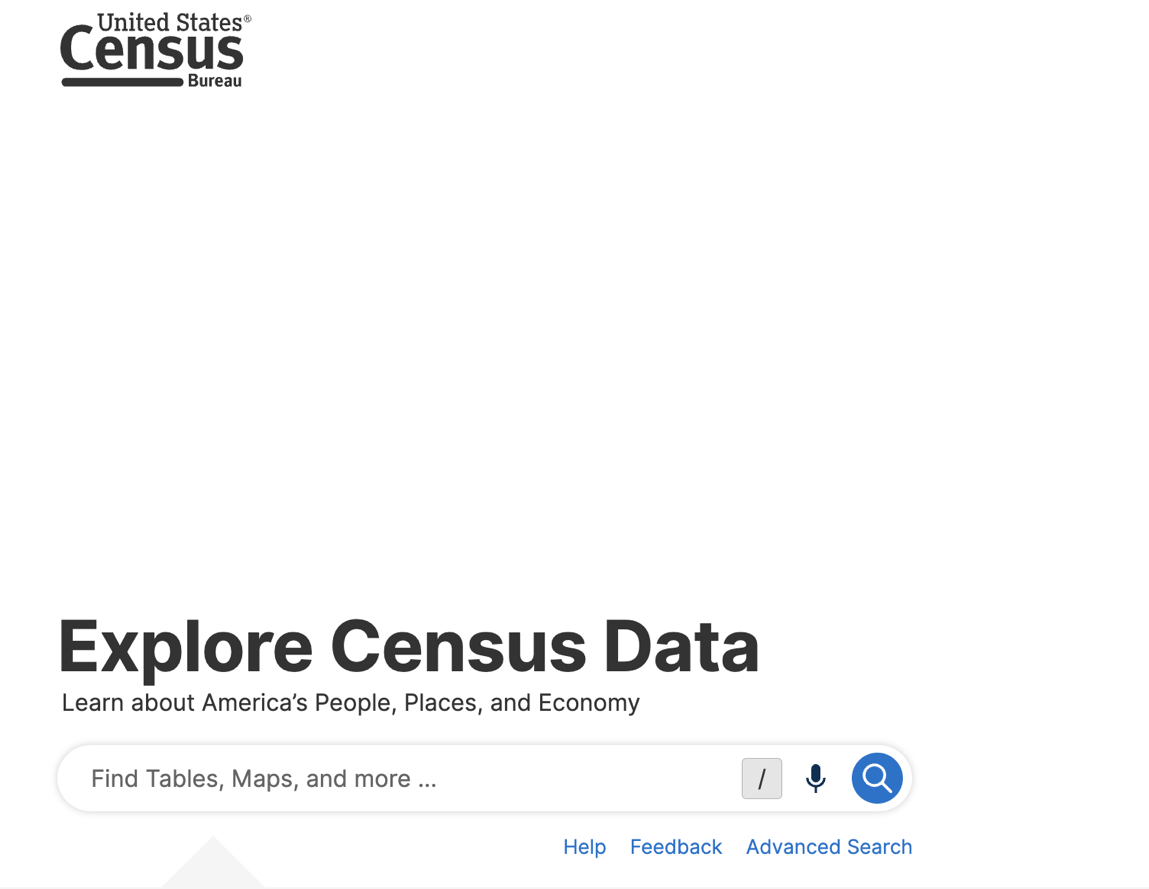


**Step 2** – Select advanced search under the search bar to display all filters.


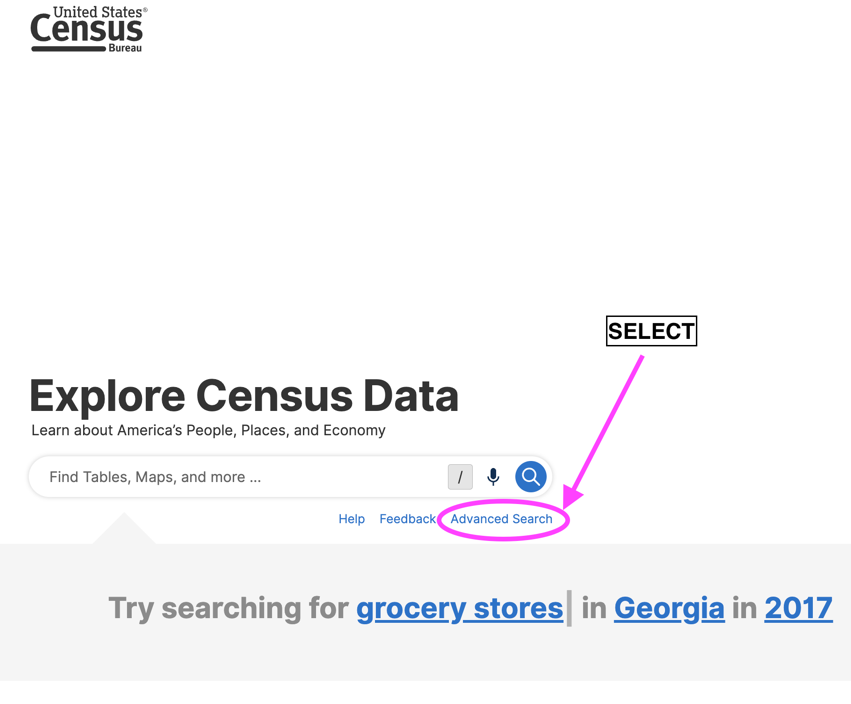


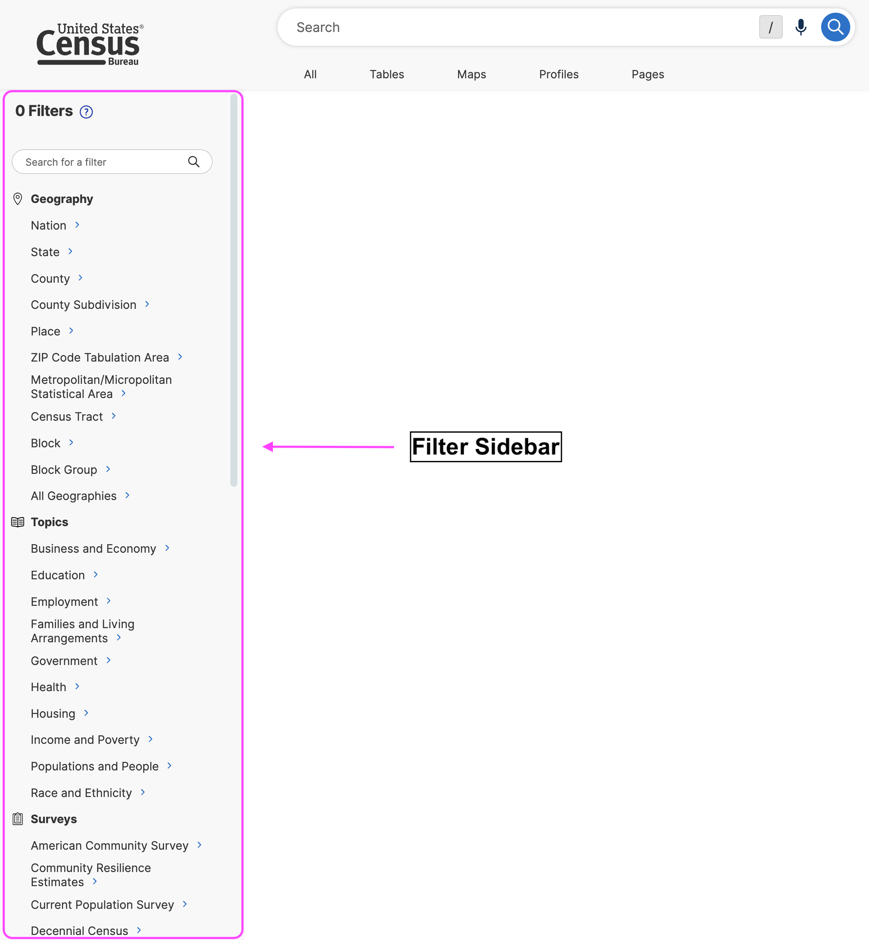


**Step 3** – Specify region by selecting state(s) or nation in the filter sidebar.


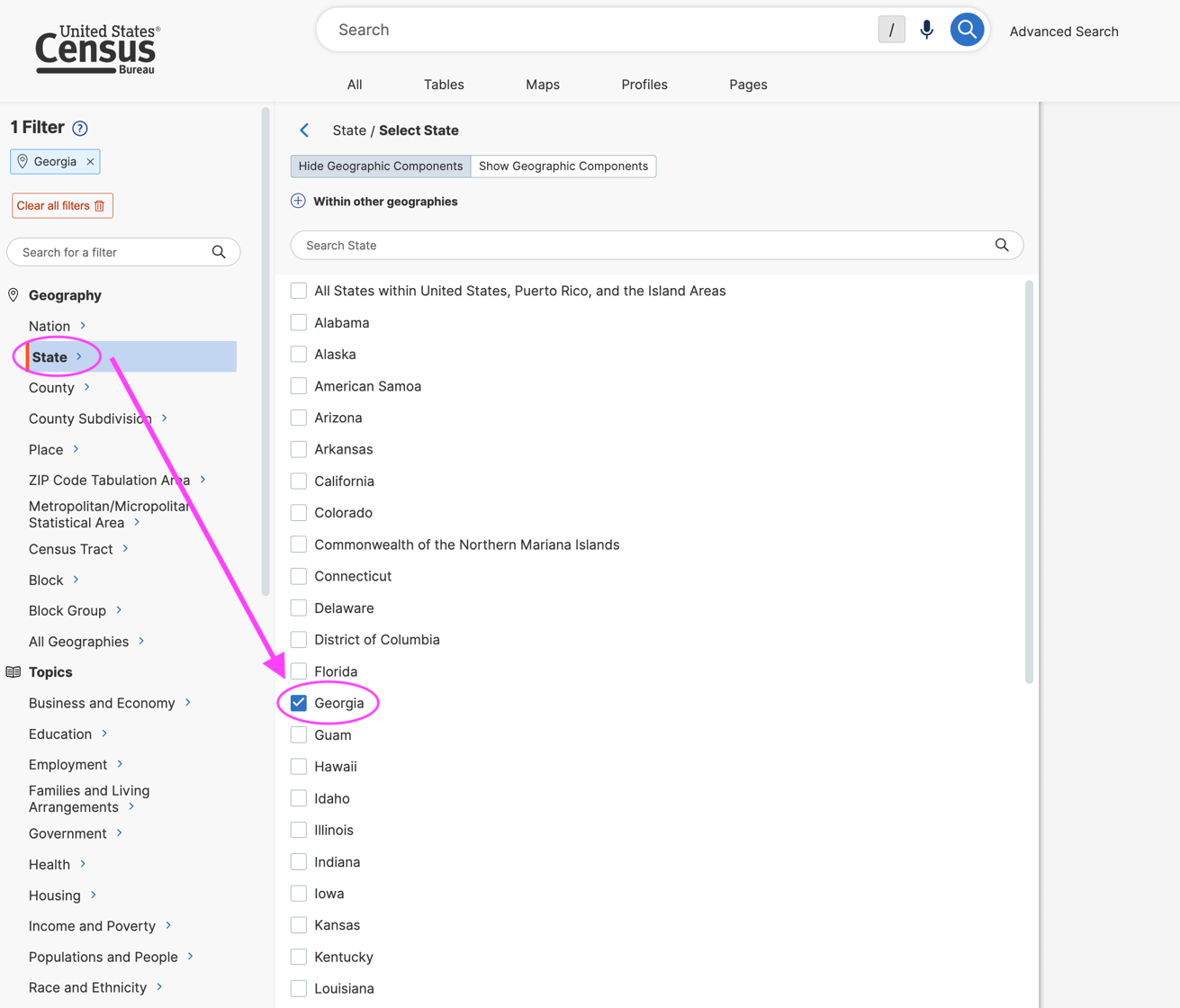


**Step 4** – Specify NAICS industry code. Use “Search for a filter” function and enter “561710.” Select the corresponding checkbox.


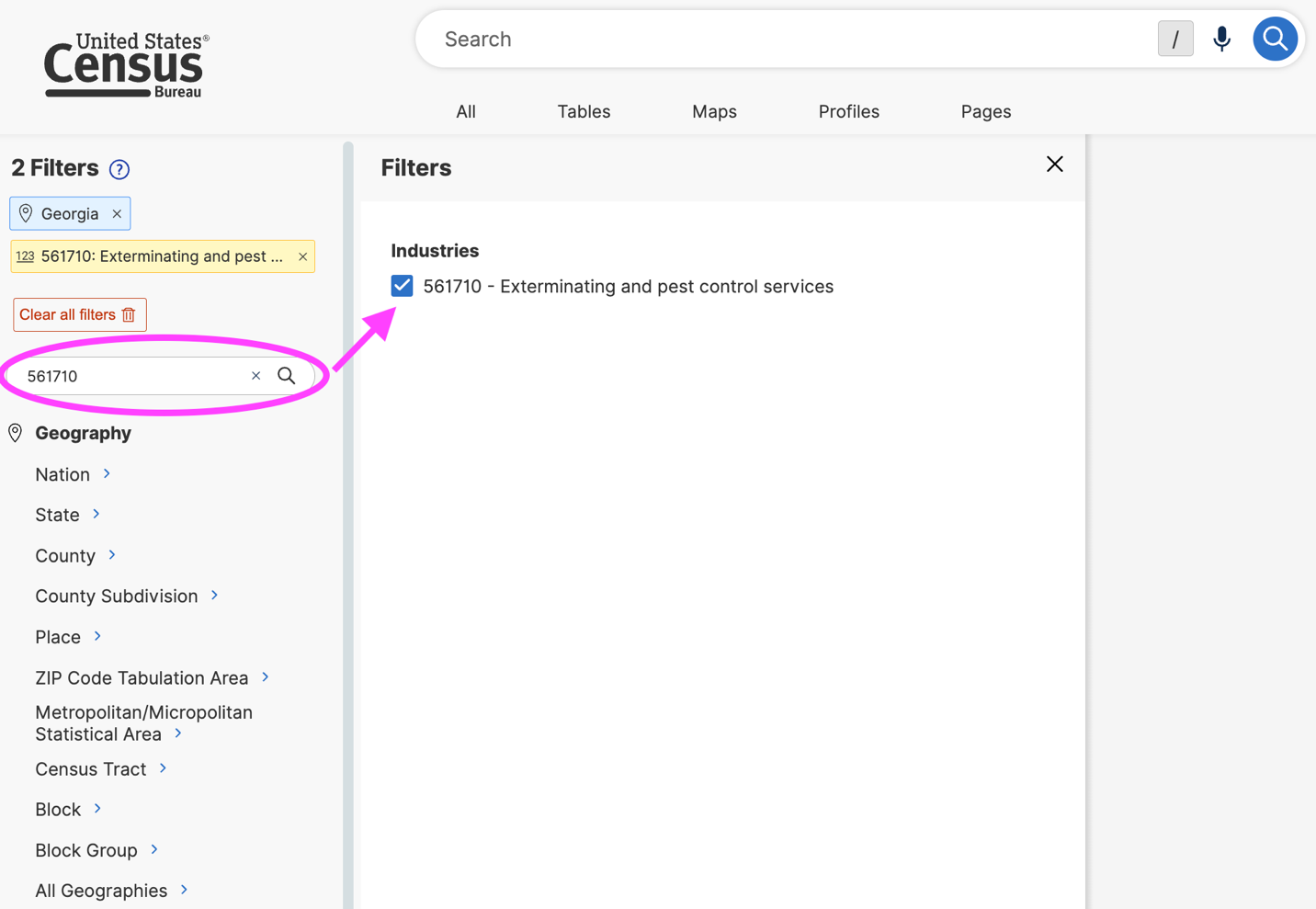


**Step 5** – Select Economic Census under the Surveys section in filter sidebar. Click on Core Statistics and then select “All Sectors: Comparative Statistics for U.S., States, and Selected Geographies (Previous NAICS Basis).”


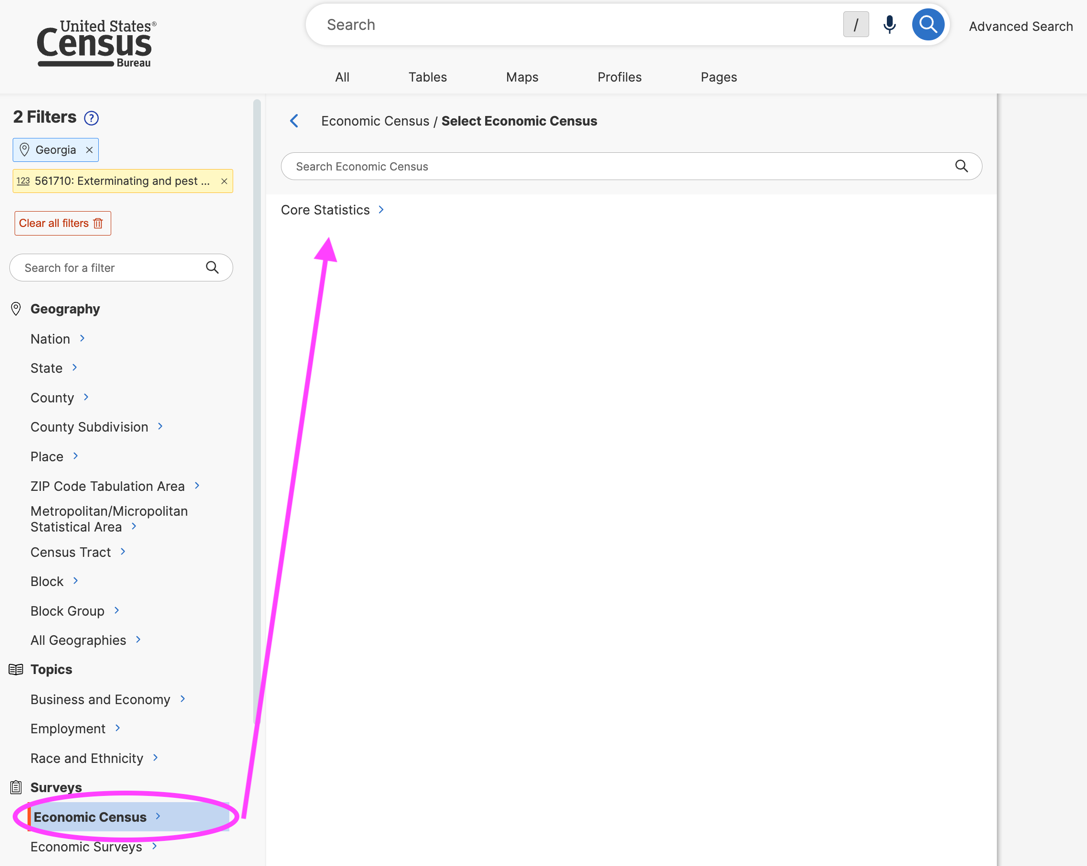


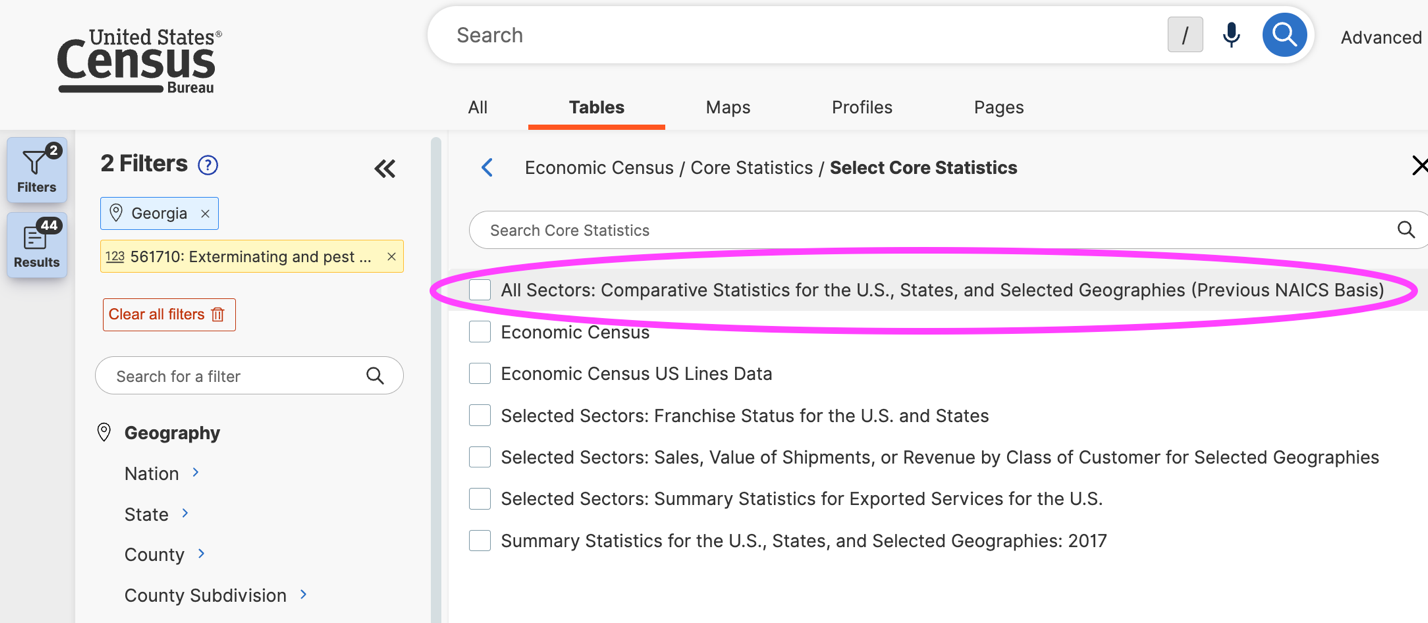


**Step 6** – Select Table “EC1700COMP.” This table provides both 2012 and 2017 values.


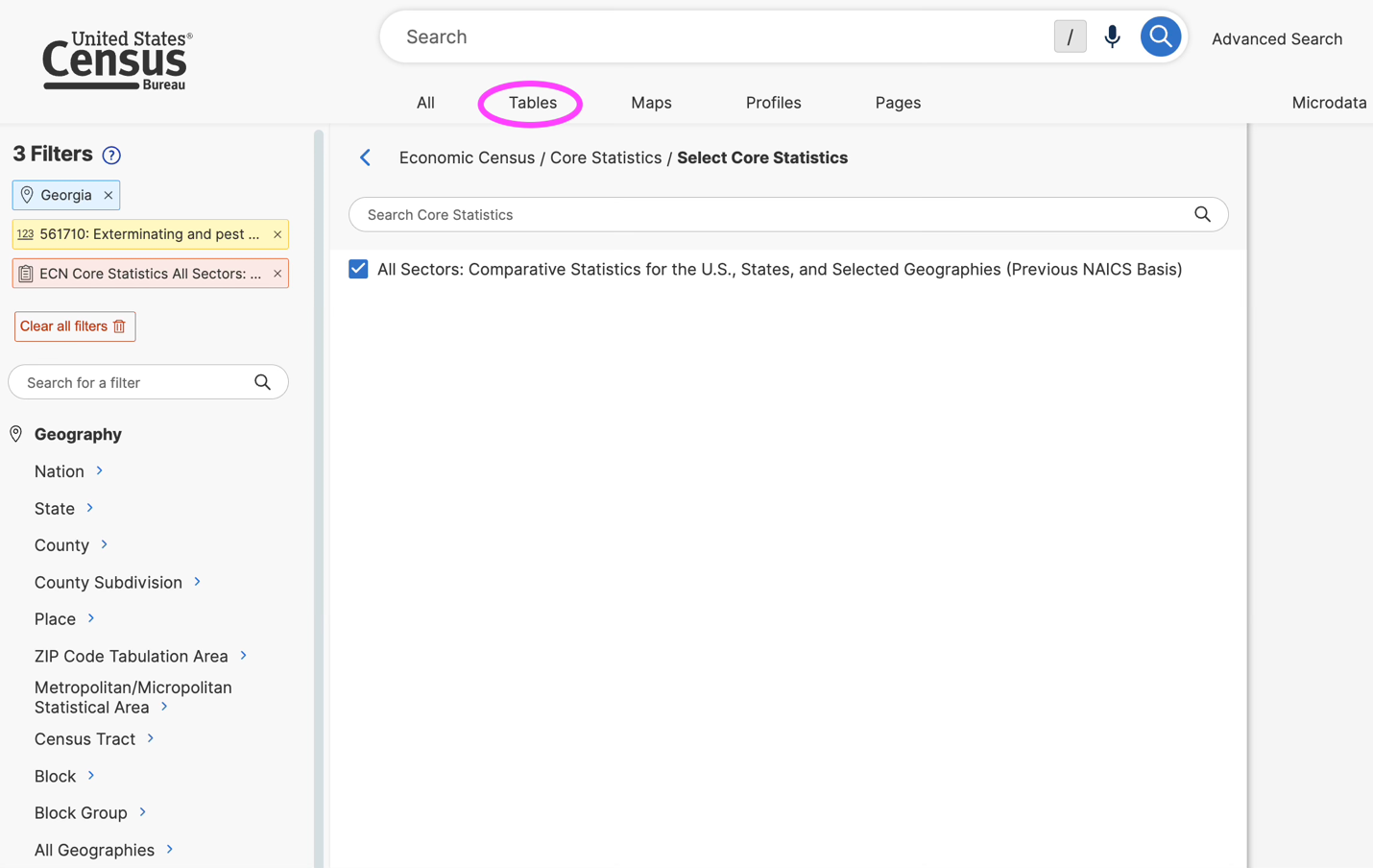


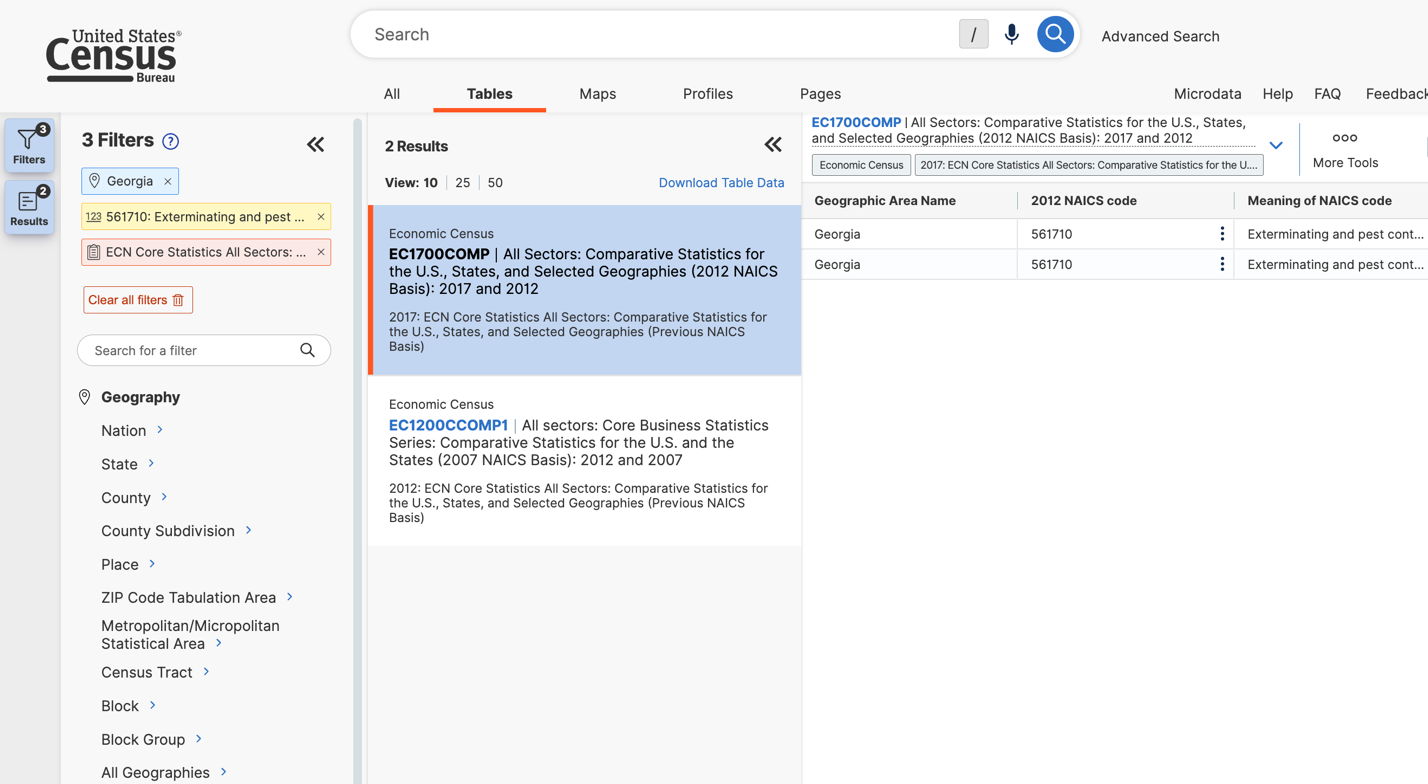

Supplement: toae029_suppl_Supplementary_Material_S6 [file toae029_suppl_supplementary_material_s6.docx]
