# Supplementary material for: Estimate of the revenue and economic contribution of the professional pest management industry in Georgia, United States
Source: J Econ Entomol. 2024 Feb 25;117(2):601–8. doi: 10.1093/jee/toae029 (PMC11011618; doi:10.1093/jee/toae029)

Publications containing economic statistics for Georgia, USA PPMI. The first and third screenshots correspond to issues of the Georgia Pest Control Association (GPCA) newsletter, *Profile*, for 2012 and 2002, respectively. The 2002 statistics were reprinted from the Summary of Losses from Insect Damage and Cost of Control in Georgia published by the Department of Entomology, University of Georgia, which is no longer available online. The second screenshot with the green background is a leaflet distributed by GPCA in 2020.


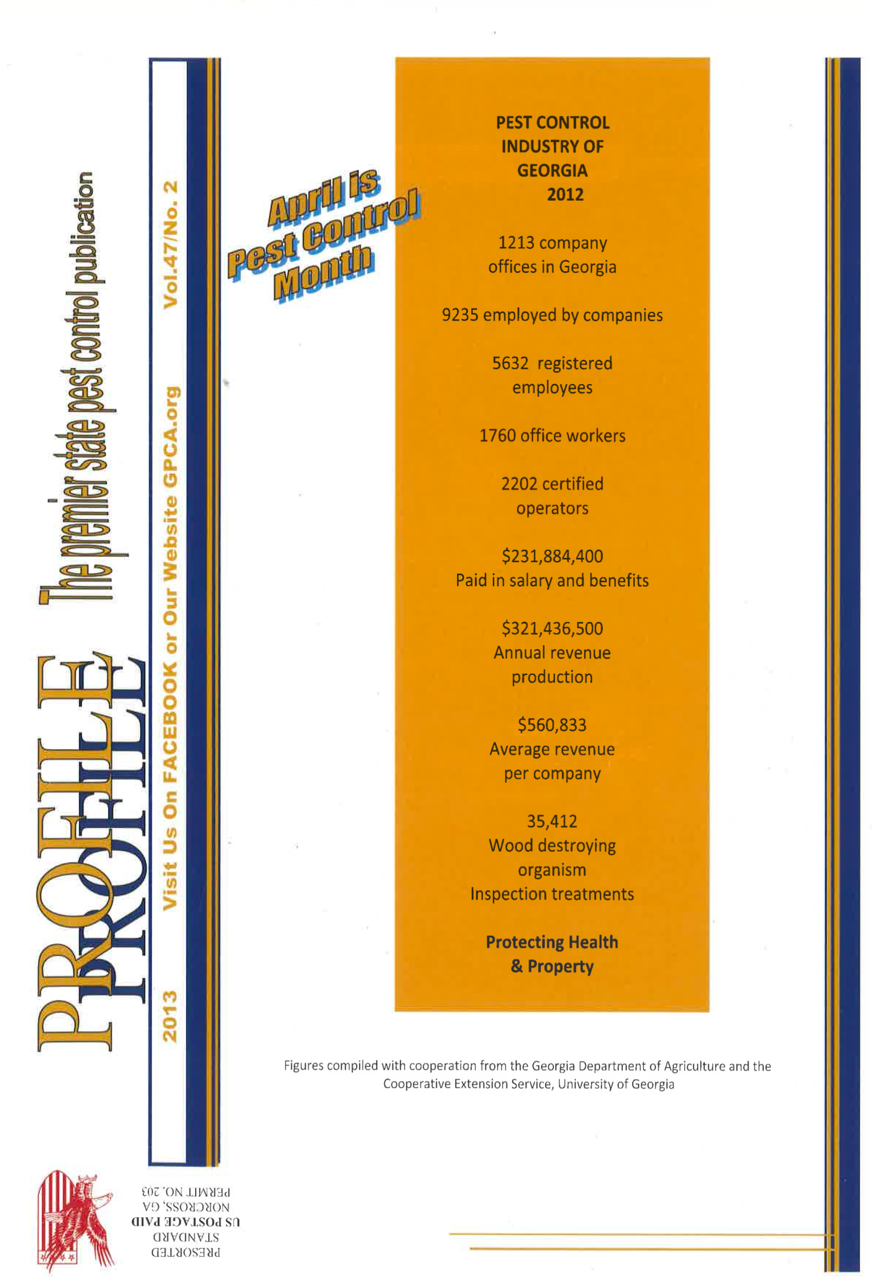

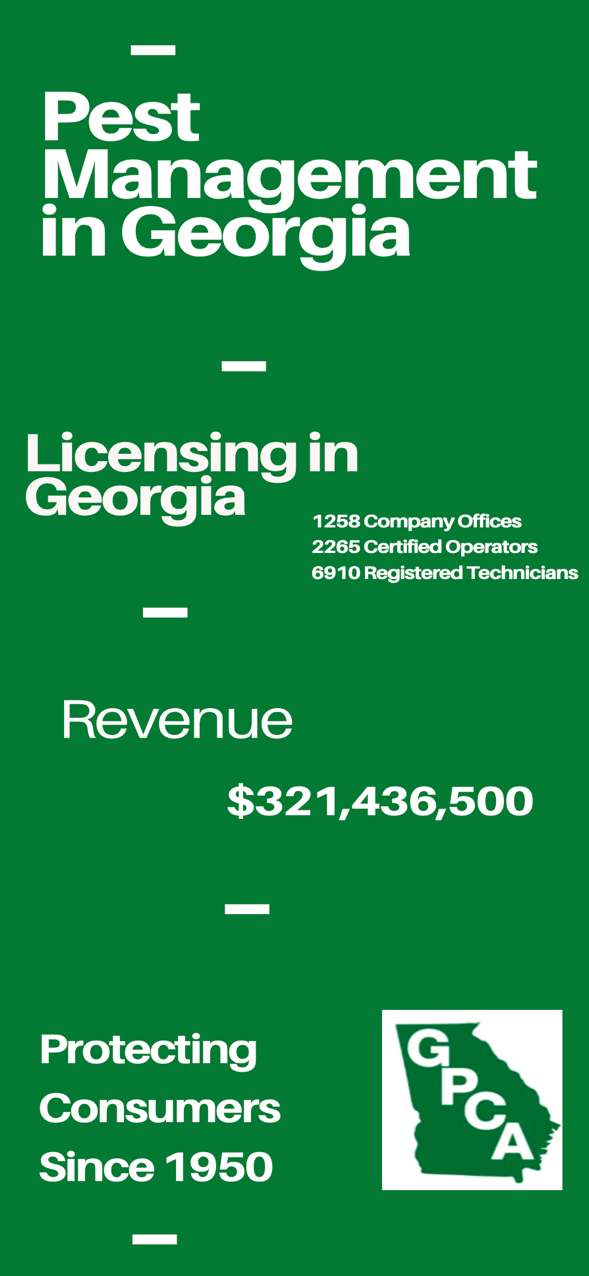


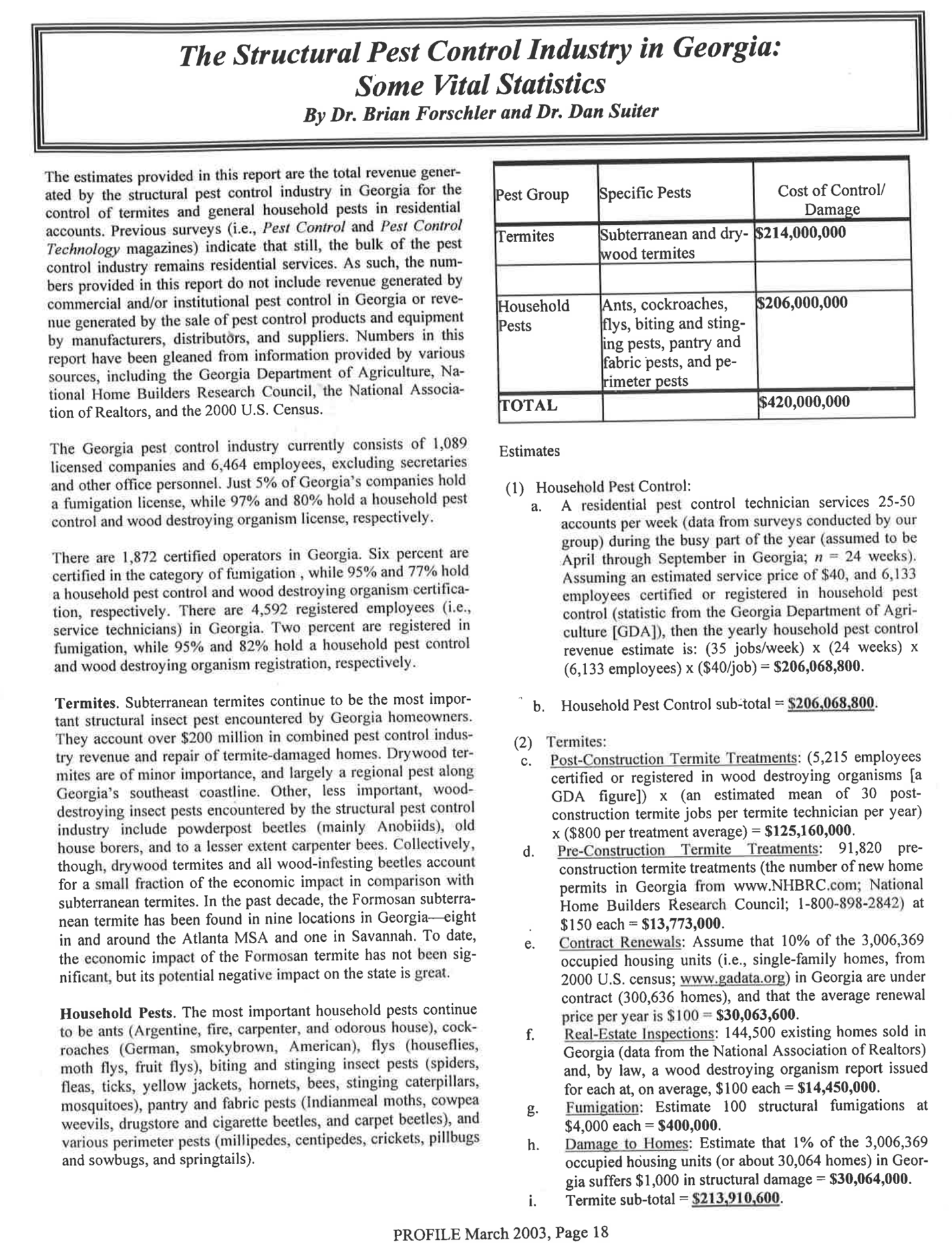

Supplement: toae029_suppl_Supplementary_Material_S7 [file toae029_suppl_supplementary_material_s7.docx]
