# Supplementary material for: Estimate of the revenue and economic contribution of the professional pest management industry in Georgia, United States
Source: J Econ Entomol. 2024 Feb 25;117(2):601–8. doi: 10.1093/jee/toae029 (PMC11011618; doi:10.1093/jee/toae029)
Supplement: toae029_suppl_Supplementary_Material_S8 [file toae029_suppl_supplementary_material_s8.docx]

The following flowchart shows the hierarchal path of the North American Industry Classification System (NAICS). This system is utilized by most governmental statistical agencies to classify and organize data (ie. employment, revenue, and number of establishments). This process aids in appropriately accessing PPMI-specific data through these various statistical agencies.

Sector (56) – Administrative and Support Services and Waste Management and Remediation Services

**Flow Chart of the Professional Pest Management Industry**

**North American Industry Classification System**

Subsector (561) – Administrative and Support Services

Industry (561710) – Professional Pest Management Services

Industry Group (5617) – Services to Buildings and Dwellings
